# The syntactic complexity of Russian relative clauses

**DOI:** 10.1016/j.jml.2012.10.005

**Published:** 2013-06-17

**Authors:** Roger Levy, Evelina Fedorenko, Edward Gibson

**Affiliations:** a Department of Linguistics, UC San Diego, 9500 Gilman Drive #0108, La Jolla, CA 92093-0108, USA; b Department of Brain & Cognitive Sciences, Massachusetts Institute of Technology, 77 Massachusetts Ave., Cambridge, MA 02139, USA

**Keywords:** Sentence comprehension, Parsing, Syntax, Memory limitations in language processing, Expectation-based processing, Russian

## Abstract

Although syntactic complexity has been investigated across dozens of studies, the available data still greatly underdetermine relevant theories of processing difficulty. Memory-based and expectation-based theories make opposite predictions regarding fine-grained time course of processing difficulty in syntactically constrained contexts, and each class of theory receives support from results on some constructions in some languages. Here we report four self-paced reading experiments on the online comprehension of Russian relative clauses together with related corpus studies, taking advantage of Russian’s flexible word order to disentangle predictions of competing theories. We find support for key predictions of memory-based theories in reading times at RC verbs, and for key predictions of expectation-based theories in processing difficulty at RC-initial accusative noun phrase (NP) objects, which corpus data suggest should be highly unexpected. These results suggest that a complete theory of syntactic complexity must integrate insights from both expectation-based and memory-based theories.

## Introduction

Human language is distinctive among the communicative systems found in nature in its infinite expressivity. To a first approximation, every utterance that a comprehender hears is one that they have never heard before. The comprehender must thus deploy finitely-represented knowledge of language in real time to analyze the utterance. A crucial aspect of this knowledge is that of syntax, which allows a comprehender to recover the meaningful relationships between words arranged in sequences that may never have previously been encountered. The cognitive effort required for the deployment of syntactic knowledge is, however, highly variable across sentences and across words within a given sentence. In many cases the difficulty of a given sentence is attributable to its specific syntactic properties. One key part of the central problem of sentence comprehension can thus be stated as follows: what major cognitive constraints govern the deployment of syntactic knowledge to achieve understanding in real time?

It has long been known that one major cognitive constraint in the deployment of syntactic knowledge is that humans cannot simultaneously pursue all possible analyses of an input string (partial or complete) in a cost-free way. Hence extensive work has been done on the problem of syntactic ambiguity resolution, where a local ambiguity of syntactic interpretation is subsequently resolved through the influence of one or more information sources (Bever, 1970; Ferreira & Clifton, 1986; Frazier & Fodor, 1978; MacDonald, Pearlmutter, & Seidenberg, 1994; Mitchell, 1994; Tanenhaus, Spivey-Knowlton, Eberhard, & Sedivy, 1995; Tanenhaus & Trueswell, 1995; Trueswell, Tanenhaus, & Garnsey, 1994; among many others). For example, the first three words of sentence (1) are ambiguous between readings in which the defendant is the agent or the patient of the verb “examined”:


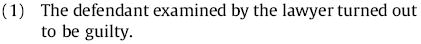


For native English speakers there is measurable processing difficulty during comprehension of the rest of the sentence, which rules out the agentive reading. Though there remains disagreement regarding precise empirical details in syntactic ambiguity resolution, most notably how quickly non-syntactic information sources can be utilized, whether more than one analysis can ever be simultaneously entertained (e.g., Clifton et al., 2003), and the extent to which globally incoherent analyses are considered (Tabor, Galantucci, & Richardson, 2004), considerable evidence has also accumulated demonstrating humans’ abilities in this area, and probability theory has emerged as a powerful formal framework for describing the cognitive constraints relevant in ambiguity resolution (Jurafsky, 1996).

Yet there are also well-documented processing difficulty effects which do *not* seem to arise from ambiguity in the analysis of a partial input string; we will use the term syntactic complexity to describe such cases (Gibson, 1998, 2000; Lewis, 1996; Miller & Chomsky, 1963; Yngve, 1960, inter alia). The present paper reports experiments designed to shed further light on the nature of the cognitive constraints underlying syntactic complexity, about which there is less agreement in the field. One hope is that theories of syntactic complexity in locally unambiguous contexts may be able to subsume theories of ambiguity resolution and thus lead to a more parsimonious and satisfactory theory overall (Clifton & Frazier, 1989; Gibson, 1991, 1998; Grodner, Gibson, & Tunstall, 2002; Hale, 2001, 2003, 2006; Levy, 2008). In the study of syntactic complexity, relative clauses (RCs) have played a particularly prominent role, partly because they exemplify one of the formally most complex corners of natural language syntax and play a key role in how language achieves its full richness of expressive capacity, and partly because they have been a rich source of empirical syntactic-complexity results. One of the most-studied cases is the asymmetry in processing difficulty between English subject-extracted and object-extracted transitive RCs as in (2) below, in which both the head noun phrase (NP; *the reporter* in (2)) and the RC-internal NP (the subject in an object-extracted RC, or the object in a subject-extracted RC; *the senator* in (2)) are animate, definite, and full.


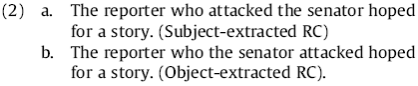


A wide range of experimental studies (Ford, 1983; Grodner & Gibson, 2005; Gordon, Hendrick, & Johnson, 2001; King & Just, 1991; Traxler, Morris, & Seely, 2002; Wanner & Maratsos, 1978, inter alia) have demonstrated that comprehension difficulty is differential for these cases: the object-extracted RC (ORC; (2b)) is more difficult than the subject-extracted RC (SRC; (2a)). These studies have also demonstrated that processing difficulty is localized: the locus of greatest processing difficulty is at the ORC verb (Grodner & Gibson, 2005). More recently, the results of Staub (2010) suggest that the onset of the subject NP in ORCs—the word *the* in (2b)—may also be a locus of some processing difficulty (a point we will return to in the general discussion of Experiment 2). Hence ORCs of the type seen in (2b) are more complex than the SRCs of the type seen in (2a), and the measurable processing difficulty associated with that complexity is localizable to two different regions within the RC. The English SRC/ORC processing asymmetry of (2) serves as an effective touchstone for describing the wide variety of theories of syntactic complexity prominent in the literature today and upon which the new research reported in this paper, on the syntactic complexity of Russian relative clauses, will bear. The remainder of this introduction provides an overview of both general and RC-specific theories of syntactic complexity. Experiment 1 and Experiment 2 each report two studies on Russian RC comprehension designed to discriminate and test the predictions of a wide range of these theories. We conclude with a general discussion of these results and their theoretical implications.

### Memory versus expectations as foundations of syntactic complexity

In broad strokes, two prominent classes of theory regarding the key cognitive constraint determining syntactic complexity can be identified: theories based on memory limitations and theories based on expectations (see Gibson & Wu (2013) for a similar summary). One such theory based on memory limitations is the Dependency Locality Theory (DLT, closely related to its predecessor, the Syntactic Prediction Locality Theory: Gibson, 1998, 2000), according to which the key operations in syntactic comprehension are storage and retrieval of potential elements in structural dependency relationships within a sentence, and integration of a retrieved preceding element into a structural dependency relation with the current input. On this theory, the resources involved in retrieval, integration, and maintenance of stored-element representations are limited. Thus dependency integrations are more difficult when more elements need to be integrated simultaneously, and when the retrieved elements have greater linear distance from the integration site. The DLT successfully predicts the English SRC/ORC processing difficulty asymmetry: the most integration-intensive word in either RC of (2) is the ORC verb *attacked*, with which both the preceding subject and object NPs must simultaneously be integrated; no other word in (2a) or (2b) involves more than one simultaneous integration (see Gibson, 1998, 2000 for further details).

A closely related theory is the activation and cue-based retrieval theory of Lewis and Vasishth (2005; see also Lewis, Vasishth, & Van Dyke, 2006; Vasishth & Lewis, 2006). In this theory, the representation of a sentence in real-time comprehension is an incrementally extended syntactic structure; similar to DLT, the theory’s processing bottleneck is retrieval of a preceding syntactic element or elements from this structure, with which the current input word must be integrated. Once an element is stored in memory, its activation level begins to decay, so that greater linear distance between a dependent and its governor generally increases the difficulty of the dependency integration, as in DLT. A distinguishing feature of the activation and cue-based retrieval theory, however, is that when elements of the incremental structure are accessed intermediately, they are reactivated, counteracting decay. This reactivation means that additional intervening constituents can under some circumstances facilitate rather than hinder an integration spanning long linear distances (Vasishth & Lewis, 2006). Countervailing against the facilitatory effect of reactivation, however, is Similarity-Based Interference (SBI; Gordon et al., 2001, Gordon, Hendrick, & Johnson, 2004; Lewis & Vasishth, 2005; Lewis et al., 2006; McElree, 2000, McElree, Foraker, & Dyer, 2003; Van Dyke & McElree, 2006): because memory is content-addressable and retrieval involves cue-based competition among stored syntactic elements, retrieval is more difficult and error-prone when the preceding context contains other elements featurally similar to the retrieval target. For (2), activation and cue-based retrieval, as well as other SBI-based theories, predict the English SRC/ORC processing difficulty asymmetry because in the ORC case, both *reporter* and *senator* need to be retrieved at the RC verb and associated with their appropriate semantic roles, but they interfere with one another due to their similarity (e.g., both are animate, singular, and definite). Both DLT and activation & cue-based retrieval make fine-grained predictions regarding the processing difficulty of each word in a sentence.

In expectation-based theories of syntactic complexity, in contrast, the key constraining factor is not memory but rather experience and/or generalization: structures with which individuals have more direct experience, or which they infer to be likely in a particular context given their linguistic and world knowledge, are easier to process in comprehension. In word-order frequency theories, surface orderings of word classes which occur more frequently in the input are hypothesized to be favored and thus easier to process during comprehension (Bever, 1970; MacDonald & Christiansen, 2002). In such theories, the greater processing difficulty of the ORC in (2) would be attributed to the fact that its surface word order, Object–Subject–Verb (reporter-senator-attacked in 2b), is rare in English, whereas the SRC has the ubiquitous surface word order Subject–Verb–Object. Such theories are closely related to the Tuning Hypothesis (Mitchell, Cuetos, Corley, & Brysbaert, 1995), which posits that coarse-grained structural statistics are tracked in linguistic input and used to make decisions in online comprehension, though the Tuning Hypothesis has historically been framed with respect to problems of ambiguity resolution rather than with problems of syntactic complexity. The predictions of word-order frequency theories regarding where difficulty will be observed, however, are relatively coarse-grained, not word-by-word.

Another expectation-based theory is surprisal, according to which comprehenders maintain and update fine-grained expectations regarding upcoming input at multiple levels of linguistic structure (including but not limited to syntax), and the difficulty of processing an input in the context in which it appears decreases monotonically as the input’s conditional probability increases (Hale, 2001; Levy, 2008; Smith & Levy, 2008, 2013). Surprisal is in some ways like a word-by-word instantiation of the word-order theory outlined above, but does not commit to the stance that expectations are based on superficial sequences of word categories. Rather, in many models instantiating surprisal theory rich syntactic context is taken into account (Boston, Hale, Kliegl, Patil, & Vasishth, 2008; Boston, Hale, Vasishth, & Kliegl, 2011; Demberg & Keller, 2008; Hale, 2001; Levy, 2008; Roark, Bachrach, Cardenas, & Pallier, 2009), so that, for example, RC-internal word order expectations might in principle be completely different from the expectations arising in independent clauses with superficially similar word order, depending on the grammatical properties of RCs in the language in question. Surprisal can account for the overall difference in English SRC/ORC comprehension difficulty because among transitive RCs whose head noun and RC NP are both full, definite NPs, SRCs are much more common than ORCs (Hale, 2001; Levy, 2008; Reali & Christiansen, 2007; Roland, Dick, & Elman, 2007); hence, the overall surprisal of the ORC is higher than that of the SRC. Surprisal is less effective, however, at predicting *where* processing difficulty in ORCs is localized: it predicts that the processing penalty is paid at the onset of the RC NP, which disconfirms the possibility that the RC is subject-extracted (see discussion in Grodner & Gibson, 2005; Levy, 2008). As mentioned earlier, the results of Staub (2010) suggest that there is in fact a processing cost at this point, but the bulk of experimental data point to the RC verb as the primary locus of ORC processing difficulty (Gordon et al., 2001; Grodner & Gibson, 2005; Staub, 2010). However, word-order theories and surprisal gain some additional degree of support from studies indicating that more frequent types of ORCs are in fact easier to process (Gennari & MacDonald, 2009; Gordon et al., 2001, 2004; Reali & Christiansen, 2007; Traxler et al., 2002; Warren & Gibson, 2002). As one particularly striking example, Reali and Christiansen (2007) found that among English RCs with pronominal RC NPs, ORCs (such as *the woman who you called*) are actually more frequent than SRCs (such as *the woman who called you*). Reali and Christiansen also found that among RCs of this type it is ORCs, not SRCs, that are read more quickly.

A third expectation-based theory is the Entropy Reduction Hypothesis (ERH; Hale, 2003, 2006). In the ERH, the entropy (Cover & Thomas, 1991; Shannon, 1948) of the distribution of possible structural completions of the sentence at any point in incremental processing is a quantity of fundamental interest; it is posited that processing difficulty ensues when a word causes a large drop in this entropy. According to the analysis of Hale (2003), the ERH successfully localizes processing difficulty at the verb of English ORCs: the point immediately following any common noun is high-entropy because common nouns are often recursively postmodified; the possibility of recursive postmodification yields a high-entropy distribution over sentence continuations. The RC verb rules out this infinity of possible NP postmodifications, and thus yields a large drop in entropy. In SRCs, in contrast, the verb follows the word *who*, which does not admit the possibility of recursive postmodification, so that the drop in entropy induced by the RC verb is much smaller.

**Fig. 1:**
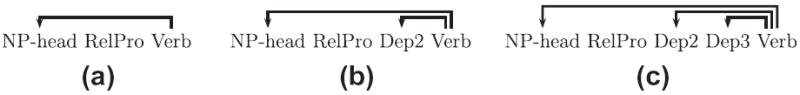
Increasing the number of dependents preceding the verb in an RC. For English, SRCs generally match configuration (a) (with additional dependents appearing only postverbally), whereas ORCs match configuration (b).

Although memory- and expectation-based approaches are aligned in predicting the general pattern of English ORCs being more difficult than SRCs, they differ in their specific empirical predictions regarding word-by-word processing difficulty, both for RCs and other constructions. These differences can perhaps be cast into sharpest relief when syntactically constrained contexts are considered: cases where the preceding context of a sentence sets up an expectation that some syntactic category X will be encountered in upcoming input, but precisely when X will appear and what word will instantiate it remain unknown until it is encountered. In these cases, expectation-based and memory-based theories make close to opposite predictions regarding the effect of processing difficulty of X as a function of the number of X’s preceding dependents. For memory-based theories, the more material appearing in the input before X is encountered, the greater the burden placed on memory and hence the harder X should be to process when it is encountered. For expectation-based theories, in contrast, additional material can on average only help the comprehender sharpen their expectations regarding the location and identity of X; this additional material should thus in general facilitate processing of X when it appears.^1^ One set of circumstances in natural language syntax in which this occurs ubiquitously is in the processing of verbs whenever they are not obligatorily clause-initial, so that the number of dependents of the verb that appear preceding it may vary.^2^ English relative clauses are such a syntactically constrained context: once the initiation of the RC is cued by the relative pronoun, the comprehender knows that an RC verb must appear (Grodner & Gibson, 2005; Levy, 2008). In the SRC, this verb appears immediately after the RC onset (Fig. 1a), at which point the comprehender was as yet uncertain as to whether the RC is subject-extracted and has seen only one of the arguments of the RC verb. In the ORC, in contrast, this verb appears after the comprehender knows that the RC is not subject-extracted and has seen two arguments of the RC verb (Fig. 1b). Thus the comprehender should have a stronger expectation in the ORC that the verb will appear when it does in fact appear, and should have sharper expectations regarding the identity of this verb; but at the same time needs to perform more memory retrieval operations upon encountering the verb, and these retrieval operations may be more difficult than in the SRC case.

In the case of English RCs as seen in (2), the observed pattern of processing difficulty matches the predictions of memory-based theories such as DLT and activation & cue-based retrieval. However, results from empirical investigation of other syntactically-constrained contexts conform in many cases with the predictions of expectation-based theories such as surprisal or the ERH, not with those of memory-based theories. As one example, Vasishth and Lewis (2006) used self-paced reading to study online comprehension of Hindi object-extracted relative clauses, as in (3) below:


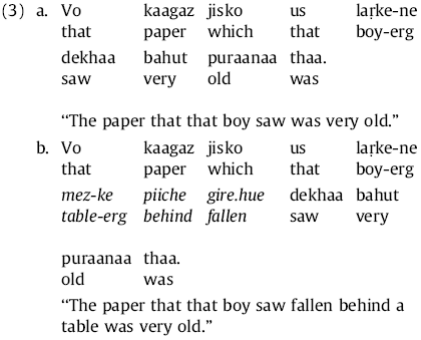


The RC verb *dekhaa* (“saw”) has more preceding dependents in (3b) than in (3a); thus memory-based theories predict greater integration difficulty. However, Vasishth and Lewis (2006) found that reading times at the RC verb were faster, not slower, in (3b) than in (3a). This finding is difficult to reconcile with the DLT: additional preverbal dependents apparently reduce, rather than increase, processing difficulty at the verb. For activation and cue-based retrieval, this finding can be explained as the additional intervening constituents reactivating the prediction for a clause-final verb set up by the RC onset and the RC-initial subject NP (Vasishth & Lewis, 2006). For the ERH, this finding could be predicted assuming that the additional preverbal dependents in (3b) render the uncertainty immediately before the RC-final verb regarding how the RC might be completed lower than in (3a). The same considerations hold for surprisal as for the ERH; furthermore, the extra preverbal dependents may give the comprehender additional predictive benefit regarding the identity of the RC verb (e.g., there are fewer eventualities that might hold with “paper” as object and “fallen behind a table” as a secondary predicate than with “paper” as object alone). Similar patterns of results have been found in comprehension of verb-final main clauses in German (Konieczny, 2000; Konieczny & Döring, 2003) and Japanese (Miyamoto & Nakamura, 2003; Nakatani & Gibson, 2010; Ueno & Garnsey, 2008); there has also been one report of such effects in English main-clause verbs following subject-modifying relative clauses (Jaeger, Fedorenko, & Gibson, 2008a).

### Syntactic complexity theories specific to relative clauses

The present experiments also bear on theories of syntactic complexity specific to relative clauses—theories which propose differences in processing complexity depending on the RC’s extraction type. Perspective Shift (MacWhinney & Pléh, 1998) proposes that the English SRC/ORC asymmetry seen in (2) may arise from a processing penalty specific to cases where the grammatical roles of the head noun in the main and relative clauses differ (cf. Gibson, Desmet, Grodner, Watson, & Ko, 2005). Universal Structural Asymmetry theories (Lin & Bever, 2006; O’Grady, 1997) propose that SRCs should always be easier to comprehend than ORCs due to the higher structural position and thus greater accessibility of the SRC’s extraction site.

### Grammatical properties of languages and disentangling theories

As described in the foregoing discussion, the syntactic complexity in comprehension of verbs in general and RC verbs in particular is an area of considerable theoretical interest in which much empirical data are available and yet fail in many cases to distinguish conclusively among competing theories. The starting point for the new studies presented in this paper is the observation that a number of potentially crucial grammatical properties tend to be confounded in the available data: the word order preference of the language investigated, the morphological richness of the language, and the construction type investigated. The clearest cases supporting memory-based theories come from studies of SRCs and ORCs in English (Fedorenko, Tily, & Gibson, 2011; Ford, 1983; Gordon et al., 2001; Grodner & Gibson, 2005; King & Just, 1991; Wanner & Maratsos, 1978), French (Holmes & O’Regan, 1981; Frauenfelder, Segui, & Mehler, 1980; Cohen & Mehler, 1996), and possibly Chinese (Hsiao & Gibson, 2003; Gibson & Wu, 2013; though see Lin & Bever, 2006; Chen, Li, Kuo, & Vasishth, submitted for publication). These studies focus on relative clauses and involve languages with relatively rigid word order, predominantly SVO, and with sparse morphological marking of grammatical roles. The clearest cases supporting expectation-based theories come from studies of Hindi (Vasishth, 2002; Vasishth & Lewis, 2006), German (Konieczny, 2000; Konieczny & Döring, 2003; Levy & Keller, 2013), and Japanese (Ishizuka, Nakatani, & Gibson, 2003; Miyamoto & Nakamura, 2003; Nakatani & Gibson, 2010; Ueno & Garnsey, 2008), languages with predominantly verb-final word order, relatively greater flexibility of non-verbal constituent ordering, and rich systems of morphological case explicitly marking the grammatical role of the main dependents of the verb (though note the results of Jaeger, Fedorenko, Hofmeister, & Gibson (2008b) supporting expectation-based processing for English main-clause verbs). Furthermore, with the exception of Vasishth and Lewis’s study of Hindi RCs and Levy and Keller’s study of German RCs, the cases supporting expectation-based theories did not investigate relative clauses.

Here we report data from the comprehension of relative clauses in Russian, which are attractive in several respects given the current theoretical and empirical landscape. Like Chinese, English, and French but unlike Hindi, German, Korean, and Japanese, the predominant word order in Russian is SVO; its relative clauses appear postnominally as in English, French, Hindi, and German. Unlike the SVO languages mentioned above, however, Russian also has a rich morphological case system which marks the grammatical roles of verbal dependents, similar to Hindi, German and Japanese but unlike English and Chinese. Furthermore, Russian word order is freer than any of the above languages: although SVO is the predominant word order, all permutations of major clausal constituents are in fact permissible. This word order freedom allows us a flexibility of experimental design unavailable in these other languages: we can completely disentangle what material intervenes between an RC onset and the RC verb both from RC extraction type and from the inventory of clausal constituents encountered within the RC as whole. As a result, studying the online comprehension of Russian relative clauses may allow us to discriminate among competing theories of syntactic complexity more effectively than has been possible thus far. In Experiment 1 we use this flexibility to tease apart the contributions of extraction type and RC word order to Russian RC syntactic complexity. In Experiment 2 we use it to parametrically vary the number of clausal constituents intervening between the head noun and the RC verb; we also compare the effects of NP argument interveners and NP adjunct interveners on RC verb processing difficulty.^3^

## Experiment 1

In this experiment we use the word order flexibility of Russian to disentangle effects of extraction type from effects of word order and dependency locality on RC syntactic complexity. Any effects of word order on RC processing difficulty within extraction type could not be accounted for purely by perspective-shift or universal structural asymmetry theories. Furthermore, our manipulation of word order will have some power to discriminate expectation-based theories—specifically surprisal and potentially word-order frequency theories—from memory-based theories. We cross extraction type (SRC versus ORC) with whether the RC-internal word order is default (VO in SRCs, SV in ORCs) or scrambled (OV in SRCs, VS in ORCs) with respect to Russian’s canonical SVO main-clause word order, as in (4) below^4^:


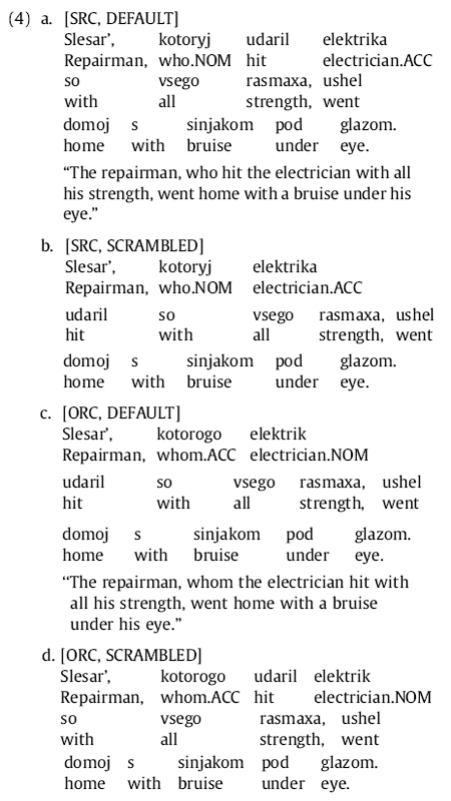


This design can also be interpreted as crossing RC extraction type with the locality (proximity) of the RC verb with respect to the relative pronoun (local in (4a) and (4d) versus non-local in (4b) and (4c)). This disentangling of verb-relative pronoun locality from RC extraction type would not be possible in languages like English with more fixed word order (e.g., Grodner & Gibson, 2005).

### Information structure and word order in Russian relative clauses

Although Russian is frequently described as a language with “free” word order, it is widely recognized among linguists who study Russian that there is strong functional motivation for the choices of different word orders in different contexts. Although a comprehensive review of the literature on this issue is far beyond the scope of the present paper, here we briefly describe issues most relevant to the present studies; the reader is referred to Krylova and Khavronina (1988), King (1995), and Bailyn (2011) for influential accounts with further references. All prominent accounts ascribe at least some degree of word order variability to information-structural considerations. Perhaps the most widely recognized characterization is the bipartite division of every Russian sentence into *theme* and *rheme*—loosely speaking, that which the sentence is about and the new information conveyed (these terms roughly correspond to *topic* and *focus* in much of both the generative and functional linguistics literatures). On the influential account of Krylova and Khavronina (1988), for example, SVO is the “default” order of simple transitive sentences in Russian, and it is generally agreed that among sentences with the most common intonational contour (so-called “non-emotive” sentences), deviations from the default word order require a context in which SVO would not satisfy the principle of the theme entirely preceding the rheme. For example,


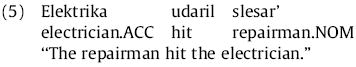


would be inappropriate in a null context, but would be appropriate as, for example, an answer to the question, “Who hit the electrician?”, which would render *elektrika* and *udaril* as part of the theme, with *slesar*’ the rheme.

Unfortunately, the vast majority of available literature in this area deals with word order in independent clauses, leaving it far less clear how such theories relate to the ordering of words within the Russian relative clause. The recent review of Bailyn (2011), for example, spends an entire chapter on word order variation in independent clauses; on the topic of subordinate-clause word order, all that is said is, “… because of the tight connection between discourse structure and word order, subordinate clauses may show less word order variation than main clauses.” Nor is this atypical of the literature. It is not even clear a priori whether the notion of “default” word order is appropriate for Russian relative clauses. Our corpus analysis and reading-time studies will turn out to be consistent with the hypothesis that for transitive subject-extracted RCs, VO would best be considered the “default”; but our results will turn out to be less clear regarding the possibility of a “default” order for object-extracted RCs. Regarding the relationship with theme-rheme or topic-focus structure, it has been informally suggested to us that the right edge of the RC may be associated with focus (Maria Polinsky, p.c.), but this issue does not seem to have been written about extensively. In our reading-time studies, the key effects are located either before the right edge of the RC (Experiment 1a) or before the comprehender could know she has reached the right edge (Experiment 1b), rendering the potential role of any such right-edge/focus association unclear.

Finally, we should make a brief remark regarding the role of information structure for online processing-difficulty effects in studies such as ours. As will become clear in our experimental results, differing word orders *do* induce differing levels of processing difficulty; most notably, SRCs with OV order will turn out to be read more slowly than SRCs with VO order, or than ORCs with either order. It seems quite plausible that information structure plays a role in this result: OV order in SRCs is rare, and may, for example, be natural only when the embedded object NP is discourse-given. If this is the case, then OV order in SRCs would be all the more unexpected in the null contexts in which we present our experimental sentences. An information-structure-based explanation of the reading-time result would require a linking theory between the discourse context (in this case null), the word order encountered, and comprehension difficulty. Among the theories we have considered, memory-based and universal structural asymmetry theories do not obviously present prospects for such a linking. Expectation-based theories such as surprisal do: the discourse context is simply part of the probabilistic conditioning context, and unexpected word orders are surprising, directly giving rise to processing difficulty when evidence of the unexpected word order is encountered.

### Predictions of different theories of syntactic complexity

We now describe the predictions of each type of theory for reading these sentences. Perspective-shift and universal structural asymmetry theories predict a main effect of extraction type, with greater difficulty for ORCs than for SRCs. Memory-based theories predict an interaction between extraction type and word-order canonicity, or equivalently a main effect of locality, with greatest difficulty in the SRC scrambled and ORC default word order conditions. This differential difficulty effect should appear at the RC verb.

As a part of determining the predictions of expectation-based theories, we conducted corpus searches to tabulate frequencies of each of the four types of Russian relative clauses in the Russian Dependency Treebank, a collection of late 20th-century texts (approximately 35,000 sentences and a total of 1 million words from a mixture of genres including fiction, news, and a small amount of scientific literature) hand-annotated for dependency structure (Boguslavsky, Grigorieva, Grigoriev, Kreidlin, & Frid, 2000; Boguslavsky et al., 2002). Inspection indicated that these frequencies differed considerably depending on (i) whether the RC-internal NP is realized as a full (versus pronominal) NP, and (ii) whether the RC was introduced with a form of the relative pronoun *kotoryj*, which has distinct forms for different case/gender combinations (e.g., nominative *kotoryj* vs. accusative *kotorogo* for animate masculine nouns), or with the relative pronoun *chto*, which is case-syncretized; hence we tabulate frequency counts specific to the different possibilities for (i) and (ii).^5^ Searches were carried out with the Tregex tool (Levy & Andrew, 2006; search patterns given in Appendix A). The results are shown in Table 1.

These results reveal several patterns relevant to our study. We do not find dramatic differences between general SRC and ORC frequencies; the strongest such difference is a 2.2:1 ratio among RCs with case-marked relative pronouns and full RC NPs (the ratio of the sum of the first two versus the last two rows of column 2 of Table 1). (For comparison, the ratio found for English by Roland et al. (2007), in the parsed Brown Corpus is 7.6:1.) Finer-grained inspection, however, revealed that the external distribution of RCs with the case-syncretized relative pronoun *chto* differed across RC extraction type: whereas SRCs occurred in otherwise-typical contexts, e.g.:


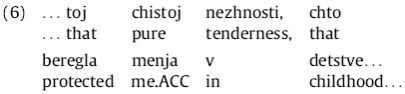


In ORCs initiated by *chto*, the head was almost invariably inanimate and extremely semantically light, e.g., *vse, chto*… “everything that”, *edinstvennoe, chto*… “the only thing that”, *poslednee, chto*… “the last thing that”. If comprehenders track fine-grained co-occurrences of this sort, then for contexts of the type seen in (4) they should interpret *chto* as a strong indicator that the upcoming RC will be subject-extracted; if, in contrast, they track coarser-grained statistics they may treat *chto* as a marker of an RC onset that is nevertheless relatively neutral to the RC’s extraction type (Mitchell et al., 1995). We will return to this issue in Experiment 1b.

Among SRCs with both relative pronoun types there is a strong preference for canonical, local word order (VO); among ORCs with both relative pronoun types, although both VS and SV orders inside the RC are seen, the preference is for the more canonical, less local ordering (SV). Similar to the findings of Reali and Christiansen (2007) for English SRC/ORC relative frequency, we find that these statistics are further dependent on the type of RC NP. However, there is a general tendency for pronominal RC-internal subjects and objects to appear earlier in the RC; when the search is restricted to only full RC NPs, the VS order is somewhat more common than SV order.

At the level of the RC as a whole, then, there is a clear prediction made by word-order frequency theories for SRCs: local word order (SVO) will be easier than non-local word order (SOV). The predictions for ORCs are less clear, and depend on the granularity at which word order statistics are computed. If we aggregate across all RC NP types (OSV and OVS), the prediction is that default word-order ORCs will be easier than scrambled ORCs; if we consider only RCs with full NPs, however (the type we use in our materials), the prediction is that scrambled ORCs should be easier than default word-order ORCs. At the level of the entire RC, surprisal makes difficulty predictions similar to those of word-order theories; but surprisal and the ERH also make more fine-grained predictions about word-by-word processing difficulty that are worth elaborating further. For surprisal, the first place where the difference in processing difficulty between SRCs and ORCs (favoring SRCs) could show up is the relative pronoun when it is case-marked, since the case marking indicates extraction type, but the effect would be small as the SRC:ORC ratio, at 2.2:1 (see above), is not very skewed. Additionally, RCs are a syntactically constrained context (as described in the section on Syntactic complexity theories specific to relative clauses), and more pre-verbal information regarding RC verb location and identity is available in non-local configurations (where the verb is at the end of the RC; SOV and OSV) than in local configurations (where the verb is RC-medial; SVO and OVS); and since surprisal does not assess costs for the representation storage, or retrieval memory of additional preverbal dependents, it predicts a processing advantage for non-local over local configurations at the RC verb. For the ERH, although one must be cautious in overstating the confidence of one’s predictions in the absence of an explicit probabilistic grammatical model, since Russian allows recursive postmodification of NPs one might reasonably expect the same processing advantage at the RC verb for local over non-local configurations as was argued for English RCs by Hale (2003). The ERH makes no obvious predictions regarding effects of RC extraction type or interactions with relative pronoun case marking.

### Experiment 1a

For this experiment we constructed sentence frames on the basis of noun pairs 〈N1, N2〉 whose positioning in the sentence was interchangeable, and included both (A) variants where N1 was the head noun and N2 the RC NP, and (B) variants where N2 was the head noun and N1 the RC NP. Since it is arbitrary which noun in the pair is considered N1 and which N2, we collapse across this manipulation in all analysis of data; but including this manipulation ensures that possible differences in event plausibility (e.g., if repairmen were more likely to hit electricians than vice versa in (4)) are not confounded with RC extraction type. We also included a three-word prepositional phrase (PP) at the end of the RC in all conditions, so that each RC in this experiment consisted of a one-word NP, a one-word verb, and a three-word PP. The NP and verb always were the first two words in the RC, appearing in the order determined by experimental condition. The RC-final PP prevents the RC-final comma from falling on the RC NP or verb, and also gives some hope of determining whether any results arising at the main-clause verb reflect spillover or processing difficulty at the main verb itself. (The possible role of spillover in influencing RTs on the RC verb is addressed in Experiment 2.) A sample item in its eight conditions would thus consist of the four “A” variants in (4) plus the four additional “B” variants in (7) below (note that the English translation depends only on the extraction type and the A/B variant).


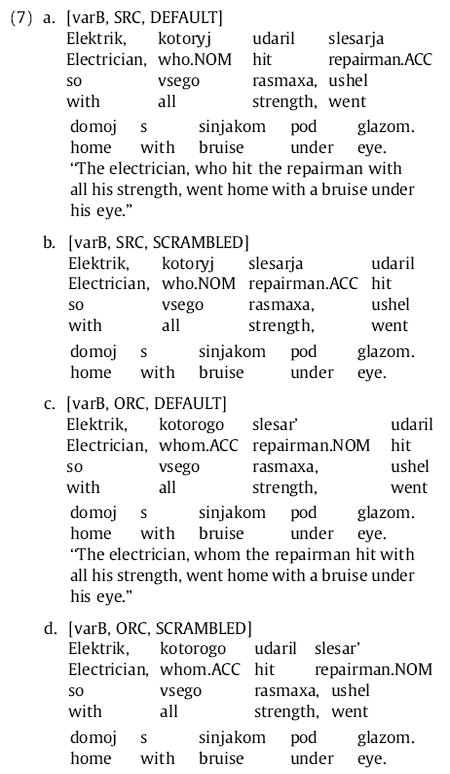


#### Participants

Sixteen native Russian speakers living in or visiting the United States participated in this experiment at the University of California at San Diego for cash compensation. None had arrived in the United States before age 13, and all reported that they continue to use Russian on a regular basis and consider it the language they are most comfortable with.

#### Materials

Thirty-two items (listed in full in Appendix C) were constructed following the pattern of (4) and (7). Each participant saw only one of the eight conditions of each item according to a Latin square design. These experimental stimuli were interleaved with 20 items from an unrelated experiment and 52 random fillers such that no two experimental sentences were seen consecutively.

#### Procedure

Sentences were presented to participants in a non-cumulative word-by-word moving-window self-paced procedure on a PC laptop computer running the Linger software (Rohde, 2005). Each trial began with a series of dashes displayed on the computer screen in place of the words in the sentence. The first press of the space bar revealed the first word in the sentence, and each subsequent press of the space bar revealed the next word in the sentence and masked the previous word. Punctuation was displayed together with the word preceding it. The times between button presses were recorded to the nearest millisecond. Each sentence was followed by a yes-or-no comprehension question probing the participant’s understanding of the content of the sentence. Written instructions in Russian were given at the outset of the experiment.

#### Results

##### Statistical analysis procedures

We used “mixed-effects”, sometimes called “multi-level” or “hierarchical”, models for all analyses. For reading-time data we used linear mixed-effects (LME; Baayen, Davidson, & Bates, 2008; Bates, 2012; Pinheiro & Bates, 2000) models, and for question–answering data we used logistic mixed-effects models (Jaeger, 2008). All our predictive factors were dichotomous, and we centered them by coding one level of the factor as −0.5 and the other as 0.5, rendering lower-order effects interpretable as in standard ANOVAs even when higher-order effects are included. Our fixed-effects model structure always reflected the factorial structure of our experiment; and we always used “maximal” random-effects structure for our theoretically critical variables—that is, by-participant and by-item random effects with the same specification as for our fixed effects (since all our manipulations were both within-participants and within-items). Using maximal random-effects structure means that our analyses make the same assumptions about participant- and item-specific sensitivities to experimental condition as in traditional ANOVAs, and ensures that the analyses are not anti-conservative with respect to the question of whether our data suggest that the effects of theoretical interest would generalize to new participants and items (Barr, Levy, Scheepers, & Tily, 2013). We present *p* values computed by treating the *t* statistic resulting from LME analysis as approximately normally distributed (justified for datasets of our size; Baayen et al., 2008). Analyses were carried out using R’s lme4 package (Bates & Sarkar, 2008).

Raw reading times were analyzed as follows, unless otherwise specified. Recordings in any region above 5000 ms or below 100 ms were discarded, means and standard deviations were then computed for each region in each condition, and any measurement more than four standard deviations above the mean was discarded. These procedures resulted in loss of 0.80% of data. The remaining measurements were then subjected to mixed-effects analyses; data from both correctly-answered and incorrectly-answered trials were included. Error bars in graphs represent standard errors of by-subject means. In-text descriptions of reading-time results are limited to regions of theoretical interest.

##### Comprehension accuracy

Table 2 shows comprehension accuracy as a function of experimental condition; no main effects or interactions are statistically significant. We see a numerical but non-significant interaction with highest accuracy in the SRC default-order and ORC scrambled conditions. This pattern could be viewed as a main effect of locality, with higher accuracy in the local conditions.

##### Reading times

We treated each of the first nine words of the sentence as its own region; an example is given in (8) below:


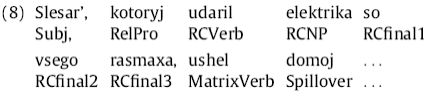


**Fig. 2:**
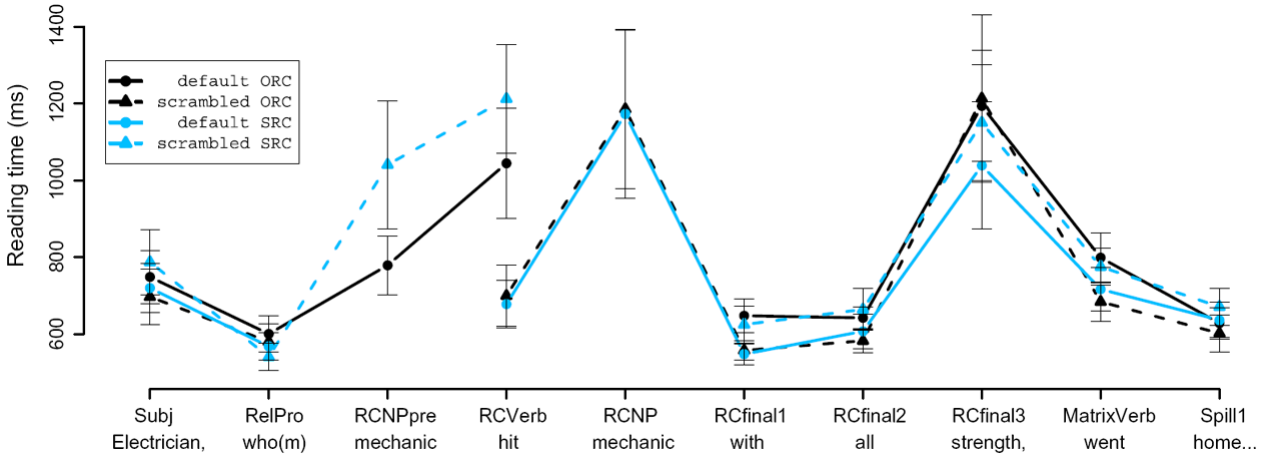
Reading times in Experiment 1a. The RCNP region appears twice because it is preverbal in the non-local conditions and postverbal in the local conditions.

Note that the RC NP is treated as a single region for the purposes of statistical analysis, regardless of its order in the sentence. This allows us to interpret effects of word order and its interaction with RC type on RC processing difficulty. Fig. 2 shows average reading times for each of these nine regions of analysis. There is a non-significant trend for the relative pronoun to be read faster in SRC than in ORC conditions. The interaction between RC type and scrambling is significant at the RC Verb (*p* < 0.001), the RC NP (*p* < 0.05), the first two of the RCfinal regions (both *p* < 0.01), and the matrix verb (*p* < 0.025). Pairwise comparisons indicate that nominative RC NPs in ORCs are read faster RC-initially than postverbally (*p* < 0.025) but that accusative RC NPs in SRCs are not (*p* = 0.296). We also conducted pairwise comparisons of RC NP reading times in the two local conditions, finding that preverbal accusative RC NPs in SRCs are read marginally slower than preverbal nominative RC NPs in ORCs (*p* = 0.059), but that in the post-verbal position there is no significant difference between the two (*p* > 0.8). At the RC verb we see a large interaction between extraction type and word order, with faster RTs in the SRC default and ORC scrambled conditions (the two local conditions). We also see a qualitatively similar but numerically much smaller interaction throughout the RC-final PP and onto the main-clause verb, with the exception of the final word in the RC, which shows a related numeric pattern (non-scrambled SRC RTs lowest) but without significant differences across condition.

We also conducted an aggregated analysis of mean per-region RTs starting at the relative pronoun and ending at the main verb. This analysis found no main effect but a highly significant interaction (*p* < 0.001).

##### Discussion

The key patterns observed in this study are (a) non-local RC configurations (ones in which the RC NP occurs preverbally) are consistently disfavored in both structural frequencies and processing difficulty, with processing difficulty peaks in the non-local configurations seen on the RC verb; and (b) a processing advantage for preverbal realization of nominative NPs in ORCs which is absent for accusative NPs in SRCs. The first pattern gives support to theories including the memory-based DLT, decay-based theories, and SBI, which predict retrieval at the RC verb to be the key factor in determining processing difficulty, with greater difficulty in the non-local conditions. As described in the section in the introduction on Memory versus expectations as foundations of syntactic complexity, the ERH is also likely to predict this locus of processing difficulty.

The second pattern merits more detailed discussion. Although memory-based theories such as DLT could predict why preverbal RC NPs are read more quickly overall than postverbal RC NPs, since only the latter can immediately be integrated with the governing RC verb, it is not clear how such theories on their own would predict the differential effects of RC NP order, since the linear dependency relationships being processed at the RC NP depend only on RC NP position and not on RC extraction type. Syntactic expectations under surprisal, however, can help clarify matters: as seen in Table 1, it is quite unlikely that an SRC will begin with an object NP, but not at all unlikely that an ORC will begin with a subject NP. Thus the fact that reading times on preverbal RC NPs are higher for SRCs than for ORCs condition is predicted by surprisal. An additive combination of surprisal and DLT integration cost (such as that used by Demberg & Keller, 2008, 2009) might thus predict a pattern similar to that seen here. An alternative possibility is that both the higher RC-verb and higher RC-NP RTs in the local conditions might reflect spillover from processing difficulty on the first (and, for the postverbal RC NP, possibly the second) word of the RC. On this interpretation, RC NP processing times critically indicate a processing *penalty* for preverbal accusative NPs in SRCs that is absent for preverbal nominative NPs in ORCs, and it is only spillover from the immediately preceding word (greater when that word is the open-class RC verb than when it is the closed-class relative pronoun) that leads to similar processing times for the accusative NPs in pre-verbal versus postverbal position.

Although the word-by-word predictions of surprisal for verb processing times were not met—it was the local verbs, not the non-local verbs, whose reading times were shortest—at a coarser granularity, in reading times across the RC and the main verb, the predictions of surprisal as determined by the frequencies of the different RC types, limited to full-NP RCs, were met: VO SRCs were read fastest overall, followed by VS ORCs, then SV ORCs, and finally OV SRCs. Additionally, RTs at the RC NP are consistent with the predictions of surprisal: a penalty is paid at RC-initial accusative NPs, which are highly unlikely among SRCs, but not at RC-initial nominative NPs, which are not so unlikely for ORCs (Table 1). Note that there was no main effect of RC extraction type in the whole-RC aggregated RT analysis, contravening the predictions of the Perspective Shift and Structural Subject Preference theories.

### Experiment 1b

We now report the results of a second experiment, 1b, with a design fundamentally similar to that of Experiment 1a and whose results largely corroborate the results of Experiment 1a. The present experiment was conducted previously to Experiment 1a, which corrects what we see as several design limitations of the present experiment. Nevertheless, the present experiment’s results are of interest because (i) it was conducted in Russia and thus has greater ecological validity (Experiment 1a was conducted among native Russian speakers living in the USA); (ii) it has a larger number of participants; and (iii) it includes a manipulation of whether a case-marked or case-syncretized relative pronoun is used to initiate the RC, which allowed us to test the extent to which comprehenders track fine-grained event probabilities, as may be predicted by expectation-based theories (see the corpus study results presents earlier; and recall that Experiment 1a used only case-marked relative pronouns). This experiment follows a design similar to that of Experiment 1a, but with several differences. The most theoretically crucial difference is (iii) above, that Experiment 1b includes case-syncretized relative pronouns as well as case-marked relative pronouns. The remaining differences are design limitations. First, unlike in Experiment 1a, thematic roles are not counter-balanced across RC extraction type. This may play a role in some of the differences in the details of the results that we find between Experiments 1a and 1b. Second, we violated prescriptive Russian orthography in omitting delimiting commas at the left and right edges of the relative clauses. Fortunately, there is no evidence in our results that suggests our participants did not rapidly adapt to this and process our sentences in overall similar ways as in Experiment 1a.^6^ Third, we did not include RC-final prepositional phrases in this experiment as we did in Experiment 1a.

#### Participants

Forty native Russian speakers participated in Volgograd, Russia for cash compensation.

#### Materials

Thirty-two items (listed in full in Appendix D) were constructed following the pattern in (4), with the differences described above. Each item began with a main-clause subject noun immediately followed by a relative pronoun (either *chto* or a form of *kotoryj*). Next came the RC NP and RC verb, each one word and appearing in an order determined by experimental condition. The main-clause verb immediately followed, after which appeared a one-word direct object and then a two-word PP adjunct. RC extraction type (SRC or ORC), relative pronoun choice (case-marked or syncretized), and RC-internal word order (default or scrambled) were factorially manipulated. As noted earlier, the only punctuation used was a sentence-final period. The eight conditions of an item can be expressed as in (9) below (cf. (4) and (7)).


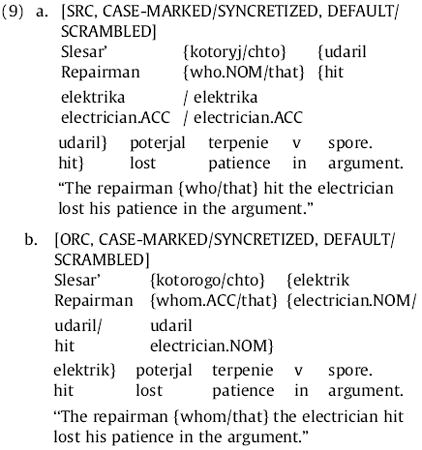


Each participant saw only one of the eight conditions of each item according to a Latin square design. These experimental stimuli were interleaved with 68 fillers such that no two experimental sentences were seen consecutively.

#### Procedure

The procedure was identical to that of Experiment 1a. The study typically took 35–45 min to complete.

#### Results

##### Statistical analysis procedures

The same analysis procedures were used as in Experiment 1a.

##### Comprehension accuracy

Question–answering accuracies for each condition are shown in Table 3. Significance results are provided in this section using by-subjects and by-subjects ANOVAs due to singular convergence of mixed logit models. We found significant main effects of RC type (*p*s < 0.001) and relative pronoun (*p*s < 0.025), and significant interaction between RC type and relative pronoun (*p* < 0.01); the primary dynamic here is that sentences containing ORCs with case-syncretized relative pronouns were by far least accurately understood. However, in separate analyses for the two relative pronoun conditions we found main effects of RC type not only in the case-syncretized condition (*p*s < 0.001) but also in the case-marked condition (*p*s < 0.01). Note that this is a different result from that of Experiment 1a; see the discussion section.

##### Reading times

Reading-time patterns were considerably different for the marked-pronoun and syncretized-pronoun conditions. Hence we plot these conditions separately. We treated each of the first six words of the sentence as its own region; an example is given in (10) below:


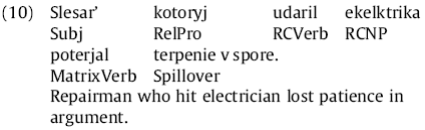


**Fig. 3:**
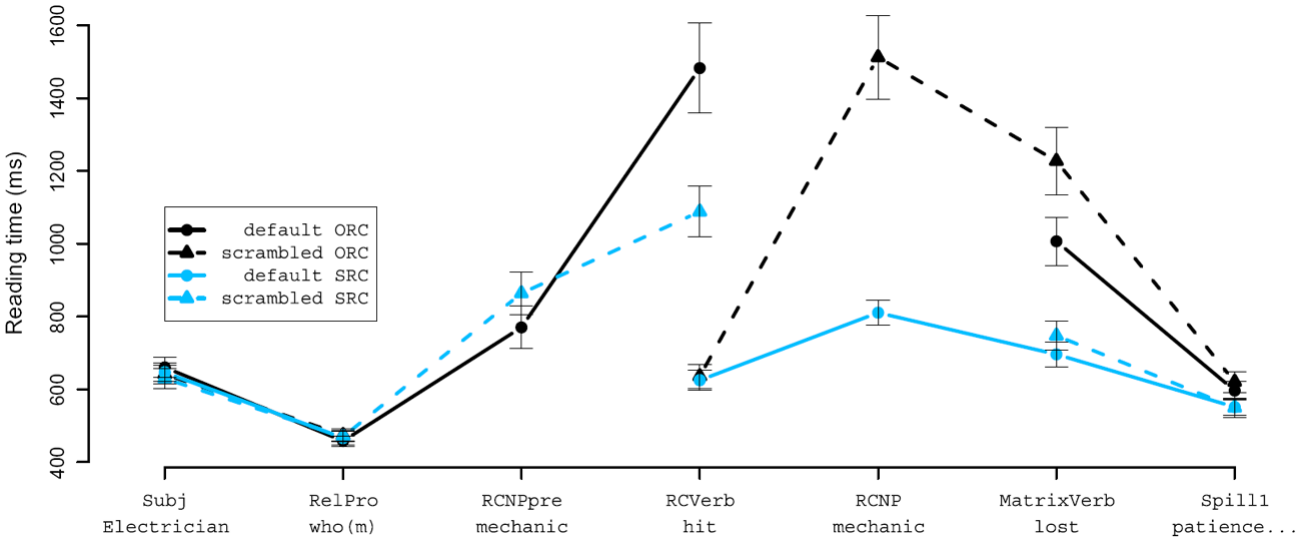
Reading times in Experiment 1b, case-syncretized (*chto*) relative pronoun conditions only. The RCNP region appears twice because it is preverbal in the non-local conditions and postverbal in the local conditions.

**Fig. 4:**
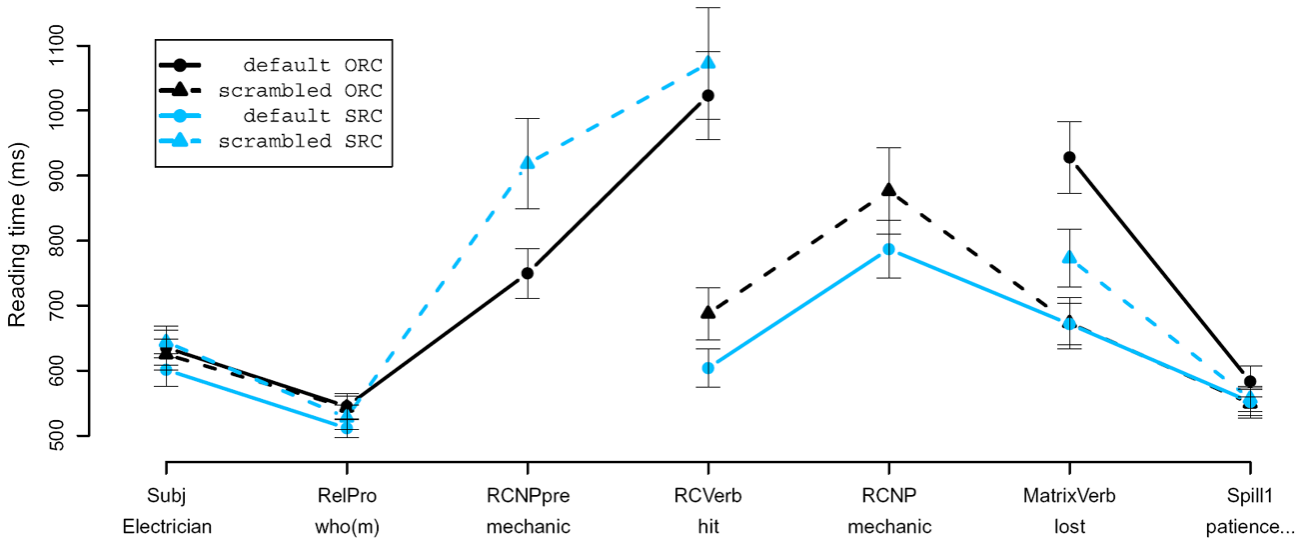
Reading times in Experiment 1b, marked (*kotoryj/kotorogo*) relative pronoun conditions only. The RCNP region appears twice because it is preverbal in the non-local conditions and postverbal in the local conditions.

Figs. 3 and 4 show average reading times for each of these six regions of analysis in the case-syncretized and case-marked relative pronoun conditions respectively (note that the *y*-axes have substantially different scales in these two figures, since the RT increases in ORCs with case-syncretized relative pronouns were so dramatic). We start with an aggregate 2 × 2 × 2 LME analysis. At the RC NP, RC verb, and matrix verb regions these analyses indicated three-way interactions (*p* < 0.001, *p* < 0.025, and *p* < 0.01 respectively), clearly driven by behavior in the two case-syncretized ORC conditions. In these two conditions (see Fig. 3), case marking on the RC NP is the first unambiguous indication of the correct grammatical function assignment to the head noun. In the case-syncretized ORC scrambled condition, where the RC NP appears postverbally, we see highly inflated reading times at the RC NP; these inflated reading times persist into the next region (the matrix verb) and to some extent to the spillover region as well. In the case-syncretized ORC default-order condition, where the RC NP appears preverbally, we see highly inflated reading times at the RC verb, which persist (though not as much in the ORC scrambled condition, leading to the three-way interaction) onto the next region, the matrix verb. Both these effects can be interpreted as an effective garden-pathing of the comprehender toward an SRC interpretation when the relative pronoun is encountered, and are consistent with the predictions (laid out in the introduction) of expectation-based theories in which fine-grained syntactic event co-occurrences are tracked: when modifying animate head nouns, *chto* is taken as a strong signal that the upcoming RC is subject-extracted. The highly inflated reading times in both these conditions can thus be interpreted as an expectation-based disambiguation penalty, though why the processing penalty is not reflected in RTs until the RC verb in the default-order condition remains unexplained.

Because the case-syncretized ORC conditions pattern so differently from the remaining conditions, we also analyzed the four case-marked conditions on their own. (Inside the RC the case-syncretized SRC conditions patterned indistinguishably from the case-marked SRC conditions.) At the relative pronoun we see a numerical advantage for SRCs over ORCs that did not reach statistical significance. At the RC NP we see a significant main effect of word order, with faster times in the default conditions. Pairwise comparisons of RC NP reading times in the two extraction conditions indicate that accusative RC NPs are read more slowly preverbally than postverbally (*p* < 0.05), but that nominative RC NPs are read marginally more quickly preverbally than postverbally (*p* = 0.054). We also conducted pairwise comparisons of RC NP reading times in the two locality conditions, finding that in the preverbal position accusative RC NPs in SRCs are read significantly slower than nominative RC NPs in ORCs (*p* < 0.01), but that in the postverbal position there is no significant difference between the two (*p* > 0.1). At the main-clause verb, we see marginal main effects of extraction type (SRCs faster than ORCs, *p* = 0.057) and scrambling (scrambled order faster than default order, *p* = 0.058). However, these main effects were driven by the overwhelmingly strongest result, a large and highly significant interaction between extraction type and word order such that the local word orders (those in which the verb immediately follows the relative pronoun) are read faster than the non-local word orders. This interaction is seen both on the RC verb and on the main verb (both *p* ≪ 0.001), with faster RTs for local than for non-local conditions.

For the case-marked conditions we also conducted an aggregated analysis of the entire RC region (beginning at the relative pronoun) plus the main verb in which the mean RT across these regions served as the response variable of analysis (analysis procedures otherwise followed those described earlier in this section). This analysis showed no main effects of extraction type or word order, but a highly significant interaction between the two (*p* ≪ 0.001).

#### Discussion

The case-marked relative pronoun conditions of this experiment replicated one key result of Experiment 1a—an interaction between extraction type and word order at the RC verb, with faster reading times in the local conditions than in the non-local conditions. The same pattern is seen at the main verb in the present experiment, and was seen in Experiment 1a throughout the remainder of the RC and onto the main verb.

The second key result of Experiment 1a involved RC NP processing times, with what we cautiously interpreted as an expectation-based processing penalty for preverbal accusative NPs in SRCs but no such penalty for preverbal nominative NPs in ORCs. In the present experiment we see the same qualitative result, but some aspects of the numerical pattern are different: in the present experiment, we see a more complete reversal of preverbal vs. postverbal positioning on RC NP reading times: accusative RC NPs are read faster postverbally than preverbally, whereas nominative RC NPs are read faster preverbally than postverbally. These results match surprisal’s predictions more straightforwardly than do those of Experiment 1a, though we hasten to point out that Experiment 1a has a cleaner design with respect to this result in that any plausibility effects deriving from differences in thematic role assignments are balanced in Experiment 1a (which included both mappings of 〈N1, N2〉 pairs into 〈head noun, RC NP〉) than in the present experiment.

Separately, the case-syncretized relative pronoun conditions of this experiment give additional evidence for comprehenders’ sensitivity to fine-grained syntactic event frequency and processing difficulty within the RC: *chto* is interpreted as a clear sign that the RC is subject-extracted. Among expectation-based theories making word-by-word predictions, surprisal predicts the ensuing difficulty (regardless of RC-internal word order) resulting when the RC turns out to be object-extracted, since the probability that the RC is a subject extraction is much higher before seeing the RC’s internal contents (recall from our corpus analysis that the vast majority of ORCs with *chto* as a relative pronoun had inanimate, semantically light head nouns, so that when *chto* introduces an RC modifying an animate head noun a comprehender using fine-grained syntactic expectations would interpret it as a strong sign that the RC is subject-extracted). It is less clear how the ERH would predict the greater difficulty observed in the ORC than in the SRC among case-syncretized conditions with preverbal RC NPs, since in both cases the RC NP’s case marking completely disambiguates the grammatical function of the head noun and it is not clear why the entropy about the rest of the sentence would be different in the two cases. This result thus gives some degree of support for expectation-based theories, most clearly surprisal—in particular regarding the magnitude of difficulty observed within the RC—though apart from the result on accusative RC-initial NPs, the question of *where* processing difficulty is first observed still seems better predicted by other theories, as we concluded in discussion of Experiment 1a.

Finally, we saw one other difference between the results of Experiments 1a and 1b: among case-marked conditions of Experiment 1b we saw lower question–answering accuracy for ORCs than for SRCs, whereas in Experiment 1a we saw no effect of extraction type on question–answering accuracy. As with RC NP reading times, the design of Experiment 1a is cleaner with respect to this result, as any potential plausibility mismatches are balanced; hence we give greater credence to its results (namely, no effects of word order or extraction type on comprehension accuracy).

### General discussion for Experiment 1

We obtained three key results consistently in both Experiments 1a and 1b. First, unlike what is typically found in English, there was no overall processing penalty for ORCs compared with SRCs. This result is problematic for perspective-shift theories, since all RCs were modifying matrix-clause subjects, and for universal structural subject preference theories. The result is consistent with memory-based theories, since difficulty for different English RC extraction types in these theories derives from differences in word order between SRCs and ORCs, but in Russian we dissociated word order from extraction type. The result is also reasonably consistent with expectation-based theories insofar as the relative frequencies of SRCs and ORCs are much less skewed in favor of SRCs in Russian than in English.

Second, processing difficulty as measured by reading times at RC verbs is greatest in non-local conditions, where the RC NP intervenes between the relative pronoun and the RC verb (i.e., SOV and OSV word orders in the RC), and least in the local conditions, where the RC verb immediately follows the relative pronoun (i.e., SVO and OVS word orders). This result is directly predicted by memory-based theories and possibly by the ERH. Universal structural asymmetry and perspective-shift theories make no predictions regarding this result. Finally, the result is problematic for word-order frequency theories and for surprisal, if the distinction between full and pronominal NPs is taken into account in determining the relevant structural frequencies (although overall whole-RC processing-difficulty pattern matches both these expectation-based theories).

Third, we found evidence of a processing penalty for preverbal accusative NPs in SRCs that was absent for preverbal nominative NPs in ORCs. In Experiment 1b this effect manifested itself quite straightforwardly: in the preverbal position accusative NPs were read faster than nominative NPs, and overall accusative NPs were read faster postverbally than preverbally but nominative NPs were read faster preverbally than postverbally. In Experiment 1a this effect was somewhat less straightforward: in the preverbal position accusative NPs were read faster than nominative NPs, and nominative NPs were read faster preverbally than postverbally, but for accusative NPs there was no difference in RTs for preverbal versus postverbal position. Of the processing theories we have examined only surprisal predicts this effect, though it remains unclear why the effect is manifested slightly differently in the two experiments.

An important limiting factor in interpreting all of the RT results in Experiment 1, however, especially the theoretically critical RT results at the RC verb, is the possibility that spillover may be affecting RTs observed at the RC NP and RC verb; it could well be the case, for example, that the inflated RTs observed on the RC verb in the non-local conditions reflect spillover from processing difficulty initiated at the RC-initial NP. Furthermore, the design of Experiment 1 does not permit us to discriminate clearly between the predictions of entropy reduction and memory-based theories, or among different memory-based theories, for RC verb RTs. Experiment 2 goes some way toward addressing these issues.

## Experiment 2

Although Experiment 1 demonstrated that self-paced reading can be used to find large and reliable differences in syntactic comprehension difficulty in Russian relative clauses of different extraction types and word orders, it did not give us full confidence in determining the precise origin sites of processing difficulty, or in distinguishing sharply between expectation-based and memory-based theories. In Experiment 2 we thus endeavor to achieve these latter goals, testing more precisely the word-by-word predictions of different theories and minimizing the possibility that spillover may obscure the origin sites of processing difficulty. We do so by parametrically varying the number of preverbal dependents in a syntactically constrained context to yield contrasts as clear as possible between the predictions of expectation-based and memory-based theories (Fig. 1). We focus our attention on subject-extracted relative clauses and consider cases where zero, one, or two constituents intervene between the relative pronoun and the RC verb. We further allow these constituents to be either arguments or adjuncts of the RC verb; we use ditransitive RC verbs so that up to two arguments are available to intervene. To maximize the possibility of distinguishing the processing difficulty associated with a given clausal constituent from spillover processing difficulty due to the onset of the preceding constituent, we make each of the constituents at least two words long. For the intervening constituents we do this by using postmodifiers; for the RC verb itself we achieve this by using a verb complex consisting of a finite verb and an immediately following infinitival verb-form (e.g., *zabyl prinesti*, “forgot to bring”). Finally, we note that some authors have suggested that reading times may tend to decrease as the position of a word within the sentence increases (Ferreira & Henderson, 1993); although we did not see such an effect in Experiment 1, any such effect would confound a result here favoring expectation-based theories. To prevent such a confound we use two-clause sentences with the RC in the second clause and, in the adjunct manipulation, place any adjuncts that are not within the RC in the first clause of the sentence, so that the linear position of the critical RC verb complex is identical across adjunct-manipulation conditions. (This is similar to the design of Jaeger et al. (2008b).) The maximally local variant of one of our items is given in (11) below; underscores indicate words presented together in a single region in self-paced reading:


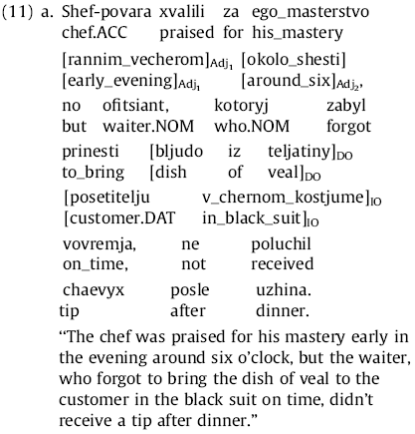


Here, the RC verb complex is *zabyl prinesti* (“forgot to bring”); its direct object (DO) is *bljudo iz teljatiny* (“dish of veal”) and its indirect object (IO) is *posetitelju v chernom kostume* (“customer in black suit”). In the argument manipulation, either the accusative or both accusative and dative arguments are fronted before RC verb, leading to the three possibilities for RC-internal word order given in (12) below:


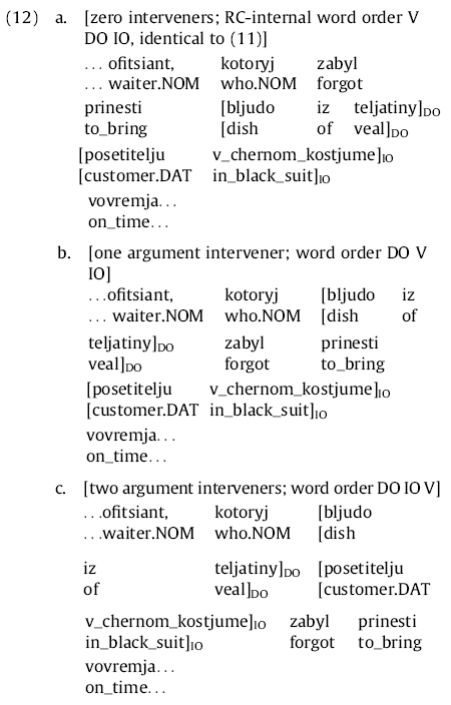


In the argument manipulation, the rest of the sentence is left the same, and the meaning of the sentence (at least in terms of predicate-argument structure) is the same in all three variants.

In the first clause of the sentence, two temporal phrases appear as well—*rannim vecherom* (“early in the evening”) and *okolo shesti* (“around six o’clock”). In the adjunct manipulation, one or both of these temporal phrases are shifted into the RC, between the relative pronoun and the RC verb complex, leading to the following three possibilities for RC-internal word order:


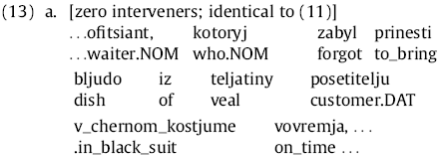



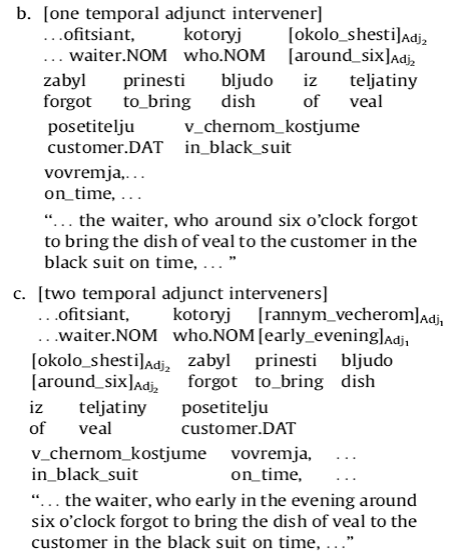


Each temporal phrase that is shifted into the RC is removed from the initial clause; thus the same set of words appears before the RC verb complex in all versions of the adjunct manipulation. Note that (11), (12a), and (13a) are all the same, hence there are five conditions in this experiment: two 1 × 3 manipulations, with the maximally local variant shared across the two.

We now go over the predictions of each class of theory for this experimental design. Perspective shift and universal structural theories of the SRC/ORC asymmetry make no predictions regarding difficulty, since all our conditions are SRCs. Memory-based theories make the simple prediction that greater numbers of interveners should lead to greater processing difficulty at the RC verb.

Intuitively, expectation-based theories predict that the presence of additional preverbal dependents intervening between the relative pronoun and the RC verb should generally facilitate comprehension of verb, because these dependents will help sharpen the comprehender’s expectations regarding where the verb will appear and which verb will be encountered, on principles similar to those described in the introduction for English RCs. The sharpening of expectations regarding verb identity should be especially pronounced for the argument manipulation: simply put with respect to (12), there are far fewer things that a waiter can do to a dish of veal with a customer in a black suit fulfilling the benefactive role than a waiter can do in general, and to “forget to bring” the dish is one of those things. As for the sharpening of expectations regarding verb location, it is plausible that once an accusative argument is seen the comprehender knows that the next constituent is less likely to be an accusative NP, hence expectations for other constituent types, including the RC verb, should strengthen; the same should happen for both dative NPs and temporal adjuncts (though the effect for temporal modifiers might be expected to be less pronounced, since multiple temporal phrases can sometimes be found in a single clause). To determine whether this reasoning is corroborated in the empirical distribution of Russian word order frequencies, we conducted a corpus study using the Russian Dependency Treebank similar to that conducted for Experiment 1. Tree-search patterns are given in Appendix A, and results of these searches are given in Table 4. Consistent with our original reasoning, adding either an intervening NP or adjunct immediately after the relative pronoun increases the conditional probability that the next clausal constituent encountered will be the RC verb. The data were insufficient for us to estimate the effect of adding a second intervening constituent except in the case of the adjunct-intervener search in the Dependency Treebank, for which the second intervening constituent raises the conditional probability of seeing a verb next even further. Thus we conclude that expectations regarding verb location are indeed likely to sharpen as the number of interveners increases. Ideally we would also estimate the effects of our intervening arguments and adjuncts on expectations regarding verb identity, but corpus data are currently far too sparse to give hope of obtaining reliable estimates.

### Experiment 2a (conducted in Russia)

#### Participants

Forty native Russian speakers participated in Kazan, Moscow, and St. Petersburg, Russia, for cash compensation.

#### Materials

Twenty items (listed in full in Appendix E) were constructed following the pattern seen in (12)–(13). For each item, the RC of interest consisted of an unambiguously nominative relative pronoun, an inanimate unambiguously accusative-marked direct object NP, an animate unambiguously dative-marked indirect object NP, a finite verb followed by a non-finite verb, an RC-final phrase, and (in the One and Two adjunct-intervener conditions) one or two temporal adjuncts. The accusative and dative NPs each had a postmodifier. Each sentence consisted of a coordination of two conjunct sentences with the end of the first sentence conjunct delimited by a comma; the RC of interest always modified the initial subject of the second sentence. The main verb region of the second sentence conjunct always immediately followed the end of the first RC. Any temporal adjunct not appearing inside the RC appeared inside the first sentence conjunct, so that the set of words appearing before the critical RC verb complex was identical across the three adjunct conditions. Each participant saw only one of the five conditions of each item according to a Latin square design. These experimental stimuli were interleaved with 60 fillers such that no two experimental sentences were seen consecutively.

#### Procedure

Sentences were presented to participants in a non-cumulative region-by-region moving-window self-paced procedure on a Dell laptop PC running DMDX software (Forster & Forster, 2003). Each trial began with a series of dashes displayed on the computer screen in place of the words in the sentence. Due to the length of these sentences, it was impossible to present them on a single line of the screen. Therefore we broke text across lines such that the critical RC verb complex was always preceded by at least one region of presentation and followed by at least one region of presentation on the same line. Participants controlled sentence presentation with a Logitech USB gamepad; the first press of a button on the gamepad revealed the first region in the sentence, and each subsequent press of the gamepad revealed the next region in the sentence and masked the previous region. The adjunct interveners, the critical RC finite and non-finite verbs, and the main verb each always appeared as individual regions; the accusative and dative NPs each appeared as either two or three regions depending on the item. Times between button presses were recorded to the nearest millisecond. Each sentence was followed by a yes-or-no comprehension question probing the participant’s understanding of the content of the sentence. The study typically took 30–40 min to complete.

#### Results

##### Statistical analysis procedures

Due to a programming error, the finite and non-finite verb in the RC were presented as a single region in Item 8. We thus excluded this item from all data analysis. Question–answering accuracies and region-by-region reading times were each analyzed in two sets of LME analyses corresponding to the argument and adjunct manipulations respectively. These analyses involved fitting one model with and one model without fixed effects of the manipulation, and using the likelihood-ratio test (with two degrees of freedom, since number of interveners is a three-level factor in each case) to assess whether the fixed effect significantly improves model fit. Both models with and without the fixed effect included “maximal” random-effects structure for both subjects and items. These analyses can be thought of as crossed random-effects analogues of traditional 1 × 3 “by-subjects” and “by-items” omnibus analyses, but which yield a single *p*-value rather than two separate p-values; and indeed traditional 1 × 3 ANOVA analyses (not reported here) yielded qualitatively similar results in all cases. Procedures within each of these analyses were otherwise the same as in Experiment 1a unless otherwise specified. As can be seen in Appendix E, there was some variability in the number of regions of presentation of the AccMod and DatMod regions of analysis, which were usually 2 and 1 regions of presentation respectively. No qualitative changes in results obtain when items with different numbers of regions of presentation are excluded from analyses.

##### Comprehension accuracy

Table 5 shows question–answering accuracy in each of the five conditions. Neither argument nor adjunct manipulation had a statistically significant effect in this measure, though there is a hint of question–answering accuracy being lower in the non-local (1 or 2 interveners) conditions than in the most local condition.

##### Reading times

For purposes of reading-time analysis we broke the RC-internal region of the sentence down as illustrated below for the two-intervener adjunct condition (13c):


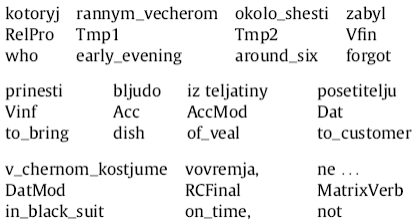


**Fig. 5:**
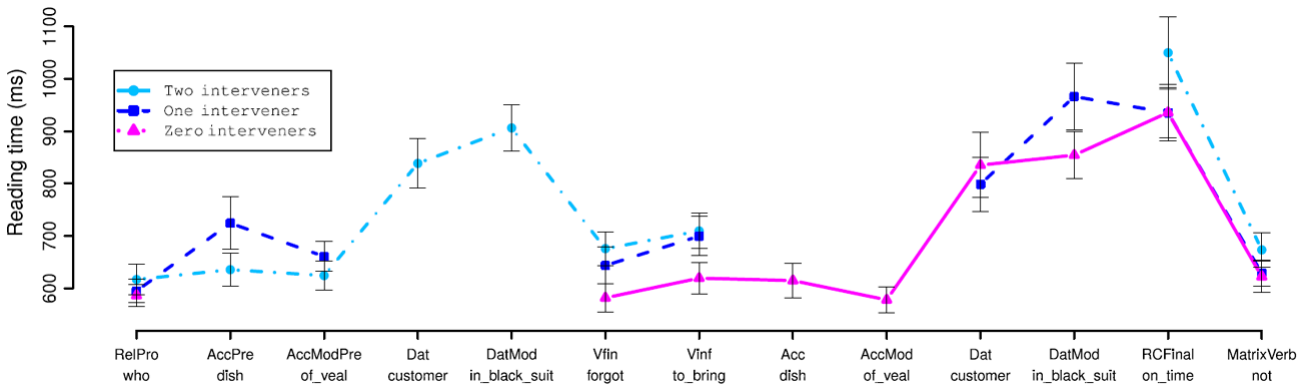
Reading times for arguments manipulation of Experiment 2a.

**Fig. 6:**
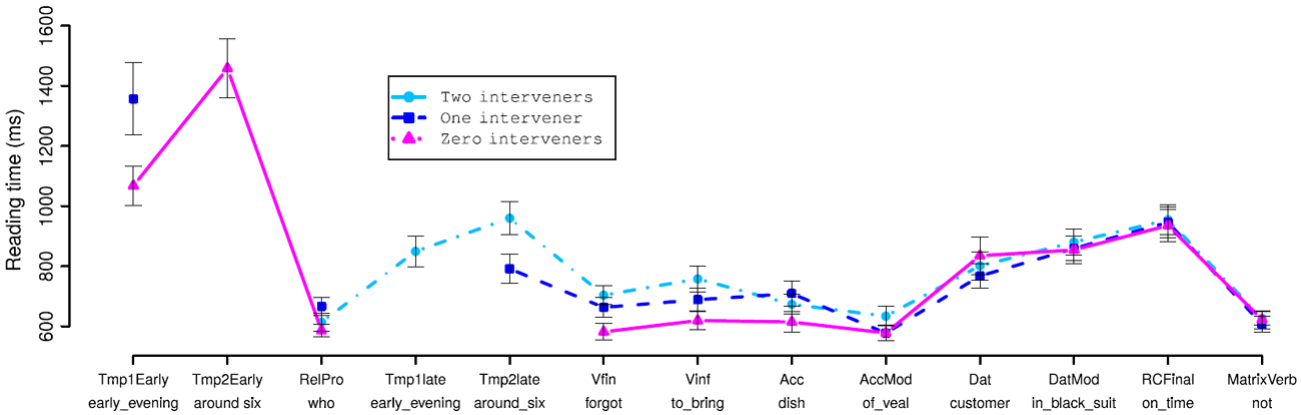
Reading times for adjuncts manipulation of Experiment 2a.

Figs. 5 and 6 show region-by-region reading times for the argument and adjunct manipulations respectively. LME analyses of the argument condition recovered significant effects of number of interveners only at the AccMod, Vfin, Vinf, and RCFinal region (all *p* < 0.05). Analyses of the adjunct condition recovered significant effects (*p* < 0.05) at all regions except for the RC-final and main-verb regions. At both the finite and non-finite verbs in the RC verb complex, reading times increase monotonically with the number of verbal dependents intervening between the relative pronoun and the RC verb, regardless of whether these dependents are arguments or adjuncts. The magnitude of the increase in verb RTs was similar for both arguments and adjuncts. This overall pattern of verb-complex RT increasing with number of interveners suggests a simple summarization in which average RT is linear in the number of interveners between the relative pronoun and the RC verb complex. To test this summarization, we fit LME models in which mean predicted RT is a linear function of the number of interveners irrespective of adjunct/argument intervener status, with random condition-specific by-participant and by-item effects, for each of the finite-verb and nonfinite-verb regions. We then compared these models against other models with the same random-effects structure but different fixed-effects structures, using likelihood-ratio tests. At both the finite and non-finite verbs, this model was significantly better than a baseline model of no condition-specific fixed effects (both *p* < 0.01), but not significantly worse than models in which number of interveners is treated as a categorical predictor and/or interacts with intervener type (all *p* > 0.4). The slopes for the linear-in-number-of-interveners models are 50.13 ms for the finite verb and 57.16 ms at the non-finite verb. Since each of these models uses only two parameters (an intercept and a slope) and is not a significantly worse fit to the data than more complex models (up to 5-parameter) models, parsimony suggests that the linear-in-number-of-intervener models are a reasonable summary of our data. Thus we can say that each additional preceding interveners increases the amount of time spent reading the RC verb by an average of 100–110 ms more time reading the RC verb complex per intervening constituent.

We also ran planned pairwise comparisons on reading times within the accusative and dative argument NPs, treating preverbal versus postverbal realization as a dichotomous variable. For these comparisons, since number of regions of presentation differed across items in the AccMod and DatMod regions, to normalize quantity of visually presented material we computed residual reading times by computing, for each region of analysis, a linear regression of reading time against (i) number of regions of presentation and (ii) total number of characters in the region of analysis. LME analyses revealed a significant effect of pre/post-verbal positioning at the AccMod region (effect size 64.21 ms, *p* < 0.01), but not at any other position.

Finally, at the final phrase of the RC (*vovremya* “on time” in Example (12)), we saw a main effect of number of interveners in the argument manipulation—driven by inflated reading times in the two-intervener condition—but not in the adjunct manipulation; this effect seems most likely a result of spillover from the RC verb complex, which immediately precedes the RC-final phrase only in the two-intervener argument condition.

Since a number of theoretically relevant effects emerge at locations where there were differences in the material being read in the previous few regions, we also conducted a spillover analysis (cf. Jaeger et al., 2008a; Mitchell, 1984; Vasishth & Lewis, 2006) accounting for reading times at previous regions. This analysis is reported in Appendix B. Crucially, no qualitative differences in RT patterns emerged from this analysis; the effect seen at the nonfinite verb became non-significant, but the effect at the finite verb remained significant, and the effect at the AccMod region became marginal.

#### Discussion

Reading-time results at the RC verb complex support memory-based theories, which predicted that increasing the number of interveners between the relative pronoun and the RC verb complex would increase processing load at the verb complex. However, the results do not clearly adjudicate between DLT and cue-based retrieval. One might say that cue-based retrieval theories predict different effects in argument- versus adjunct-intervener conditions, since the NP argument intervener, are presumably more featurally similar to the head noun than the temporal adjunct interveners and should have generated greater retrieval interference, so that the lack of clear differences between the effects of the two types of interveners favors DLT. Alternatively, one might say that it is DLT that predicts different effects from the two types of interveners, since the argument interveners uncontroversially contain discourse referents, whereas the adjunct interveners may not (depending on what counts in the theory as a discourse referent).

These results at the verb complex are unsupportive of surprisal, since the most natural prediction under surprisal would be that the additional interveners would sharpen the comprehender’s expectations regarding RC verb identity and location, and that these benefits would be realized in easier verbal processing. The results are similarly unsupportive of the Entropy-Reduction Hypothesis. The ERH’s account of the English SRC/ORC processing difficulty asymmetry relied on the fact that an immediately post-nominal position is a high-entropy position, and that discovering a verb at that point reduces entropy sharply; but this account does not predict that adding a second noun-final intervener (either a dative NP argument or a PP adjunct) would increase the processing load at the RC verb beyond the load already arising from a single noun-final intervener. However, the effect we observe at the accusative NP object—namely, slower RTs on RC-initial accusative NPs compared to post-verbal accusative NPs—is clearly consistent with the predictions of surprisal, and possibly with those of the ERH (on the assumption that the accusative NP rules out more possibilities about how the RC may unfold when it is encountered preverbally than when it is encountered postverbally).

Because of the effect observed at the accusative NP, the interpretation of the RT patterns at the RC verb complex must be tempered with a note of methodological caution: we cannot completely rule out the possibility that the differences in reading times observed on the verb complex are not the product of spillover from preceding material, especially because the material immediately preceding the verb complex differs across conditions. In the design of this experiment, these NPs were made longer (all at least three words) so as to minimize this danger of spillover. Nevertheless, since our understanding as a field of the detailed nature of spillover, including how long spillover effects can persist under a given set of experimental conditions, remains poor we cannot be sure that the length of these NPs was sufficient for the difficulty induced by their onset to be completely over by the time the RC verb complex is reached. (Additionally, we cannot be sure that the length of these phrases themselves is not itself surprising and difficulty-inducing.) The best evidence against a spillover explanation comes from two sources. First, as shown in Appendix B, even when spillover effects are partialed out of reading times the same qualitative effects of experimental manipulation are present and significant in the theoretically critical parts of our sentences. Second, reading times at and immediately following the dative NP argument are instructive: there were no significant differences on reading times within this argument, and reading times on the postmodifier of the dative argument were nearly identical across all three argument-manipulation conditions. Since this postmodifier is immediately preverbal in the two-intervener condition, it suggests that at least some part of the inflated reading times at the RC complex in the two-intervener condition is not due to any particular difficulty arising from syntactic processing of the immediately preceding constituent. We reiterate, however, that we do not view this argument as conclusive, due to our limited understanding of the nature of spillover effects in self-paced reading.^7^

Given the importance of reading time data from regions immediately preceding and following the RC verb complex, one final methodological limitation must be mentioned: while the physical presentation of sentences was designed to ensure that the critical RC verb complex did not fall near the beginning or end of a line of text, the same cannot be said for the other regions of the sentence under discussion. This concern is remedied in Experiment 2b.

### Experiment 2b (conducted in USA)

In order to address the above concern regarding physical positioning of non-critical regions with respect to line breaks, and also to test the replicability of the theoretically critical patterns observed in Experiment 2a, we ran a slightly modified version of this experiment on native Russian speakers in the United States.

#### Participants

Twenty-five native Russian speakers living in or visiting the United States participated in this experiment in Boston and San Diego for cash compensation. None had arrived in the United States before age 13, and all reported that they continue to use Russian on a regular basis and consider it the language they are most comfortable with.

#### Materials

We used the same materials as in Experiment 2a.

#### Procedure

Sentences were presented to participants in a non-cumulative word-by-word moving-window self-paced procedure on a Mac or a PC computer running Linger. Each experimental trial was presented as three lines of text. The first line break always occurred immediately before the relative pronoun. In the argument conditions, the second line break always occurred immediately after the end of the RC; in the adjunct conditions with one or two interveners, the second line break always appeared after the accusative RC-internal NP object. Unlike Experiment 2a, the postmodifiers of the accusative and dative RC-internal NPs (AccMod and DatMod) were always a single region of presentation. Procedures were otherwise identical to the preceding three experiments. The study typically took 30–40 min to complete.

#### Results

##### Statistical analysis procedures

The same procedures were used as in the previous experiments.

##### Comprehension accuracy

Table 6 shows question–answering accuracy in each of the five conditions As with Experiment 2a, neither effect of adjunct nor of argument manipulation was statistically significant. The numeric trend of Experiment 2a for questions to be answered most accurately the maximally local condition has reversed here, suggesting that there may be no true between-condition differences in accuracy.

##### Reading times

**Fig. 7:**
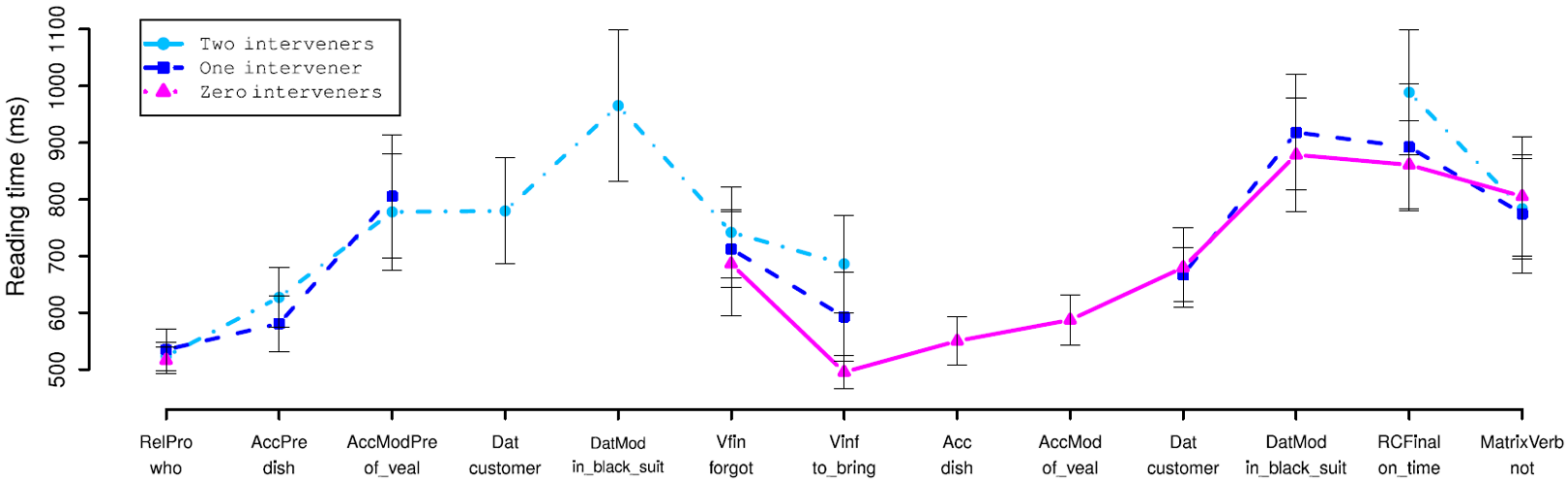
Reading times for arguments manipulation of Experiment 2b.

**Fig. 8:**
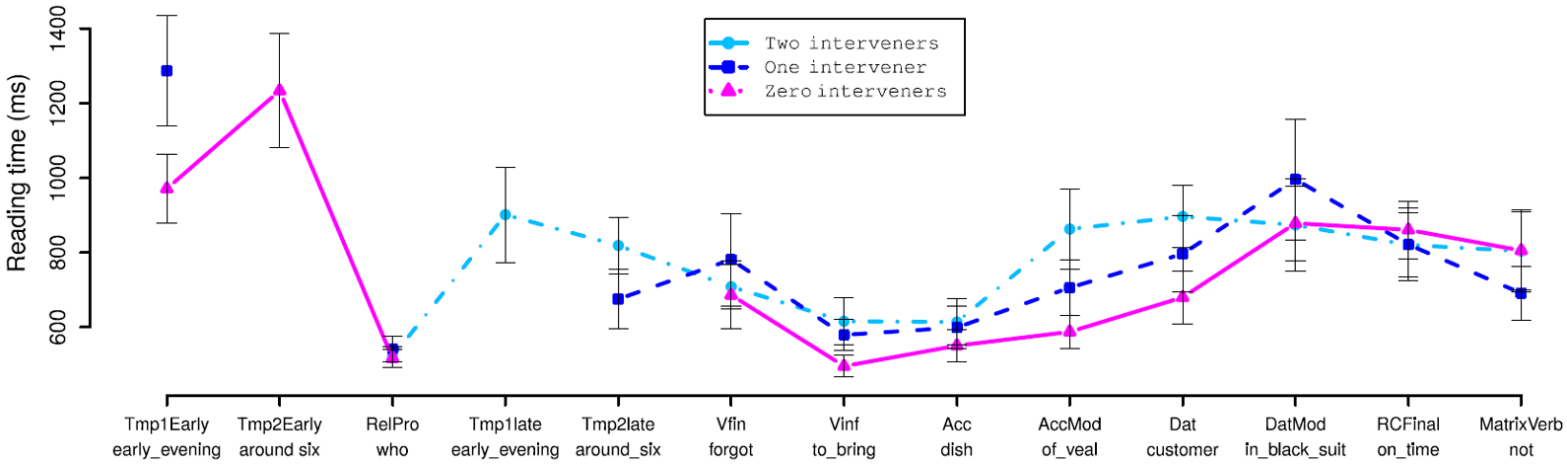
Reading times for adjuncts manipulation of Experiment 2b.

Figs. 7 and 8 show average reading times in each region for the argument and adjunct manipulations respectively. LME analyses indicated significant effects in the argument manipulation only in the AccMod and Vinf regions (both *p* < 0.01), and in the adjunct manipulation at the Tmp1, Tmp2, and Vfin regions (all *p* < 0.05), with a marginal effect at the RCFinal region (*p* = 0.097). In the argument manipulation, results look generally similar to those found in Experiment 2a: we see numerical patterns in which reading times at the RC verb complex and the final region of the RC increase monotonically with number of verbal dependents intervening between the relative pronoun and the RC verb.

To assess the evidence for the generalization from Experiment 2a that mean RTs at the verb complex increased linearly with number of intervening constituents, we again fit and compared multi-level linear models for each of the finite-verb and nonfinite-verb regions as described in Section “Reading times”. The parametric finite-verb model is significantly better than baseline (*p* < 0.01) and no model is significantly better than it, but for the nonfinite-verb region it is not significantly better than baseline (*p* = 0.47). The estimated slope in the finite-verb model is 60.66 ms; if we include the (non-significant) slope in the nonfinite-verb model of 13.05 ms, we obtain an estimate of about 74 ms additional reading time at the RC verb complex per intervening constituent, a qualitatively similar but smaller effect than in Experiment 2a.

As with Experiment 2a, we also conducted analyses of the accusative and dative NP regions with pre/post-verbal realization as a dichotomous predictor. Consistent with the results of Experiment 2a, these analyses revealed a robust effect for the AccMod region (189.91 ms effect size, *p* < 0.001); the effect was marginal at the Acc region (90 ms effect size, *p* = 0.070), but not for the Dat or AccMod regions (both *p* > 0.6).

Also as with Experiment 2a, we conducted a spillover analysis, reported in Appendix B. Crucially, none of the qualitative RT patterns changed in this analysis; both the effects at the finite verb and the AccMod region remained significant.

#### Discussion

Although with only 25 participants this experiment had less statistical power than Experiment 2a, the results largely corroborate those of the previous experiment. The main difference is that there is less evidence for differences in reading times at the finite verb of the RC in the present experiment than in Experiment 2a, though the numerical patterns at this region matched those of Experiment 2a with the exception of the two-intervener condition in the adjunct manipulation. At accusative object NPs the reading-time penalty in preverbally realized conditions is qualitatively the same as in Experiment 2a, but is significant only in the AccMod region, whereas in Experiment 2a it was significant in both the Acc and AccMod regions. The overall pattern of results in this experiment can thus be summarized as qualitatively similar to that found in Experiment 2a, except that effects here tend to emerge as reliable one region further downstream.

### General discussion of Experiment 2

The key results of Experiment 2 can be summarized as follows: in ditransitive subject-extracted RCs in Russian where both RC-internal NPs are full, processing difficulty increases monotonically with the number of interveners between the relative pronoun and the RC verb, and the RC verb complex is a locus of the inflated reading times corresponding to that processing difficulty. These inflated reading times were significant at the finite RC verb in Experiment 2b, and in both the non-finite verb and the immediately preceding finite verb in Experiment 2a. These results are predicted by retrieval-based accounts but not by expectation-based accounts. Although disentangling this effect from possible spillover effects is rather delicate (see also Appendix B), the results in the two-intervener argument manipulation seem to indicate that the RC verb complex is itself a genuine locus of processing difficulty.

One effect reliably observed in Experiment 2 is, however, more consistent with the predictions of expectation-based theories than with those of memory-based theories: the reading-time penalty paid at an RC-initial accusative object NP. We saw similar evidence for such an effect in Experiment 1. This effect is reminiscent of the result reported by Staub (2010), who compared eye movement behavior in reading of English ORCs with superficially similar complement clauses:


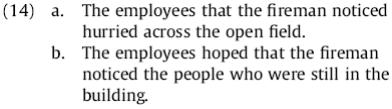


Staub found an increase in first-pass regression rate and go-past reading times on the RC-initial determiner *the* in (14a) in comparison with (14b). As noted by Staub, this result specifically confirmed a long-outstanding prediction of surprisal, according to which there should be some processing cost associated with ruling out the possibility that what is extracted from the embedded clause is the subject—a possibility absent in (14b). Notably, Staub’s data also confirmed previous findings that the ORC verb (*noticed* in (14)) is itself an independent locus of processing difficulty (e.g., Grodner & Gibson, 2005). In Staub’s data, however, signs of differential difficulty also emerged before the determiner—at the relative pronoun that, where first-fixation and go-past durations were inflated in (14a) compared with (14b), raising the possibility that the effect at the determiner is spillover from the lower expectation for the word that after a noun than after a complement clause-selecting verb.^8^ In our results for Russian relative clauses, however, no such confound exists, since the context preceding the first word in the RC after the relative pronoun is identical in all argument-manipulation conditions. Thus our results strengthen the case that, at least in some situations, encountering an NP at the beginning of an RC where it is unexpected can induce immediate processing difficulty.

## General discussion

Across four experiments with two different designs, we find two consistent patterns in reading time within Russian relative clauses varying in extraction type and word order. First, reading times at RC verbs increase monotonically with the number of dependents of the RC verb intervening between it and the relative pronoun. In Experiment 1, this pattern was seen as an interaction between the extraction type of the RC and the “defaultness” of transitive RC-internal word order: for subject-extracted RCs, the “default” verb-object order yielded faster reading; for object-extracted RCs, the “scrambled” verb-subject order yielded faster reading. In Experiment 2, the pattern was seen as a monotonic increase in reading times at RC verb complexes as the number of preverbal dependents intervening between the relative pronoun and the verb complex is increased, regardless (for the most part) of what type of preverbal dependent intervened. Although it was not clear from Experiment 1 whether this effect originates at the RC verb independently of processing difficulty associated with the onset of the RC, the results of Experiment 2 provide some degree of evidence that the RC verb is indeed an independent locus of comprehension difficulty. These results are broadly supportive of memory-based theories.

Second, in three out of four experiments there is evidence that an RC-initial accusative NP induces immediate comprehension difficulty. In Experiment 1b this effect is seen among RCs with case-marked relative pronouns as an interaction of RC extraction type and locality at the RC-internal NP; in Experiments 2a and 2b it is seen as an effect of RC-internal argument NP ordering within the accusative object NP. In all these cases, RTs are greater when the accusative NP appears RC-initially than when it appears postverbally. The exception is Experiment 1a, where the accusative NP was read numerically (though not significantly) faster RC-initially than postverbally; but even here, the interaction between word order and RC extraction type indicates that there is a processing penalty associated with RC-initial placement for accusative NPs relative to the cost of RC-initial placement of nominative NPs. This processing cost associated with RC-initial accusative object NPs is directly predicted by surprisal (and possibly by other expectation-based theories). The predictions on this front are unclear for memory-based theories: under these theories a postverbal NP is integrated with its governing verb but a preverbal NP cannot be, so the integration cost would be greater for preverbal NPs. However, the preverbal NP could be more taxing in terms of the overall representation of syntactic structure in memory: for DLT, this would be manifested in storage cost, and for activation and cue-based retrieval theories, in which incremental syntactic structure is always fully connected, it would be manifested in greater time spent structure-building to accommodate the preverbal NP than to accomodate the postverbal NP (Vasishth, p.c.). The direct support from this result for surprisal is not confounded by spillover, since it seems clear under all theories that the difficulty of immediately preceding word is consistently greater in the RC-initial conditions (a relative pronoun) than in the postverbal conditions, and since Experiment 1 suggests that this fronting penalty occurs only for accusative NPs in SRCs, not for nominative NPs in ORCs.

How the results of these experiments bear on a wide variety of prominent theories is summarized in Table 7. In Experiment 1, the lack of clearly greater processing difficulty for ORCs than for SRCs overall is damaging to perspective-shift and structural-subject-preference theories, but not to memory-based theories in which it is word order rather than grammatical function that predicts processing difficulty, or to expectation-based theories in which relative frequency of a structure determines its difficulty (word-order theories and surprisal), since the frequency ratio between ORCs and SRCs is far less skewed in Russian than in English (see the corpus study in Experiment 1). The overall processing preference for verb-local configurations observed was predicted by DLT and activation & cue-based retrieval (on the basis of locality) and by word-order and surprisal theories (on the basis of frequency). The strong bias for interpreting the case-syncretized relative pronoun *chto* as a cue indicating subject-extraction of the upcoming RC is predicted clearly by word-order theories and by surprisal, on the hypothesis that comprehenders track fine-grained co-occurrence frequencies. This pattern is what was predicted by perspective-shift and structural-subject-preference theories, but these theories do not account for why the pattern disappears when the relative pronoun is case-marked. Memory-based theories are silent on the matter, and it is not clear what prediction would be made by the ERH. The effect of number of interveners on RC verb processing times specifically is clearly predicted by memory-based theories, but contravenes the predictions of expectation-based theories. Finally, only surprisal clearly predicts the consistent processing-time penalty observed at accusative NPs placed at the beginning of SRCs (the low-frequency, non-default position).

Overall our results thus provide support for both memory-based and expectation-based theories, and by the same token are thus damaging to extant unitary accounts of processing difficulty. These results support ideas explored by researchers such as Boston et al. (2011) that “a complete theory of syntactic complexity must integrate insights from both expectation-based and memory-based theories.” The existing data might be consistent with Boston et al.’s model and with the Psycholinguistically Motivated Tree-Adjoining Grammar model of Demberg and Keller (2009), both of which include both surprisal and working-memory (retrieval for Boston et al., verification for Demberg and Keller) components of processing difficulty; it may turn out in these models that the surprisal component naturally dominates at the RC-initial accusative NP, whereas the memory component dominates at the RC verb. It is worth recalling, however, that verbs in many other language/construction combinations show expectation-based patterns that are not unambiguously predicted by memory retrieval-based theories; these cases include German main and subordinate clauses (Konieczny, 2000; Konieczny & Döring, 2003; Levy & Keller, 2013), Hindi relative clauses (Vasishth & Lewis, 2006), and Japanese main clauses (Nakatani & Gibson, 2008). This observation returns us to the original question we posed in the introduction of what properties of language/construction combinations might determine when the processing-difficulty patterns we see will most closely reflect the predictions of expectation-based theories or memory-based theories. We had raised several possibilities: the key factors might include the dominant word order of the language, the morphological richness of the language, and/or the construction type investigated (most notably whether relativization is involved). Based on our present results in Russian, we can reasonably conclude that morphological richness on its own is not a key factor. Russian is much closer to German, Hindi, and Japanese than to English in its morphological complexity, and if anything should probably be said to be more morphologically complex than German insofar as it has six cases as compared to German’s four (the comparison with Hindi and Japanese is more difficult as the distribution and functional role of case marking in these languages overlaps significantly with that of prepositions in European languages). Given that Vasishth and Lewis (2006) demonstrated expectation-based processing patterns in relative clauses for verb-final Hindi, it seems reasonable to suspect that the dominant word order of a language plays a key role in determining the syntactic complexity of relative clauses in that language. One generalization that might profitably be pursued is that the verb-medial languages tend to exhibit the general patterns predicted by memory-based theories, whereas verb-final languages tend to exhibit the general patterns predicted by expectation-based theories. Vasishth, Suckow, Lewis, and Kern (2010) provide collateral evidence for this generalization, finding that native German speakers maintain more accurate expectations for upcoming sentence structure through multiple center-embeddings in German than native English speakers do in English. This generalization is not exceptionless—Vasishth and Drenhaus (2011) have recently found evidence for integration cost effects in German when memory load is made extremely high, and Jaeger et al. (2008a) reported anti-locality effects in English at matrix-clause verbs—but it may serve as a useful rubric to guide further research on memory and expectations in syntactic comprehension. Another possibility for generalizing the present results would be to hypothesize that, as suggested by Vasishth and Drenhaus, “expectation plays a dominant role only when working memory load is relatively low” (a similar suggestion was also made by Gibson, 2007), though the question would remain as to why the working-memory threshold seems to be higher for verb-final languages like German and Japanese than for verb-medial languages like English and Russian. (A corollary of this discussion is that empirical research on RC comprehension in verb-initial languages is sorely lacking and could be of considerable theoretical value.)

Overall, these experiments underscore the value of cross-linguistic empirical breadth in advancing our understanding of both the syntactic complexity of relative clauses—a topic of theoretical interest in its own right—and more generally the interplay between memory and expectations in online sentence comprehension. The patterns observed in the study of speakers’ comprehension of sentences in their native languages are clearly emergent from a combination of the universal cognitive capacities of our species with contingent facts about the language in question. When study is restricted to a single language, however, it is impossible to discern which of these patterns are universal and which are language-contingent. Although no single theory yet explains why we see precisely the memory-based and expectation-based patterns in the circumstances we do, expanding the scope of inquiry across languages raises prospects for clarifying this picture and thereby advancing our fundamental understanding of online language comprehension. In cases of ambiguity resolution, our understanding has already benefited considerably from a broader cross-linguistic view (Brysbaert & Mitchell, 1996; Cuetos & Mitchell, 1988; Cuetos, Mitchell, & Corley, 1996; Desmet, Brysbaert, & de Baecke, 2002; Gibson, Schütze, & Salomon, 1996; Gibson, Pearlmutter, & Torrens, 1999; Mitchell & Brysbaert, 1998). With the present studies we hope to contribute to similar advances in our understanding of syntactic complexity.

## Figures and Tables

**Table 1:** Results of Experiment 1 corpus search for frequencies of Russian subject- and object-extracted RCs in default and scrambled word order. See text for further discussion of fine-grained differences between distributions of case-marked and case-syncretized SRCs and ORCs.

RC word order	*kotoryj/kotorogo* (Case-Marked)		*chto* (Case-Syncretized)	
	All constituents	Full NPs only	All constituents	Full NPs only
SRC, VO	154	147	17	11
SRC, OV	9	4	2	0
ORC, VS	42	41	9^a^	8^a^
ORC, SV	74	29	14^a^	6^a^

a Head nouns overwhelmingly inanimate and semantically light; see main text for further discussion.

**Table 2:** QA accuracy for Experiment 1a. Numbers in parentheses are standard errors.

	Default	Scrambled
SRC	0.91 (0.19)	0.82 (0.17)
ORC	0.87 (0.20)	0.85 (0.15)

**Table 3:** QA accuracy for Experiment 1b. Numbers in parentheses are standard errors.

	nscr	scr
Marked subj	0.86 (0.21)	0.86 (0.20)
Marked obj	0.80 (0.17)	0.76 (0.25)
Syncretized subj	0.88 (0.19)	0.84 (0.23)
Syncretized obj	0.68 (0.21)	0.63 (0.27)

**Table 4:** Syntactic conditional probabilities of verb given preceding RC-internal context for argument and adjunct interveners in Experiment 2.

Event and conditioning structure	Support	Probability
*p*(V∣*kotoryj*)	1574	0.573
*p*(V∣*kotoryj*, NP)	51	0.627
*p*(V∣*kotoryj*, Adjunct)	325	0.769
*p*(V∣*kotoryj*, Adjunct, Adjunct)	27	0.852

**Table 5:** Question–answering accuracy in Experiment 2a. Numbers in parentheses are standard errors.

	Zero interveners	One intervener	Two interveners
Arguments	0.72 (±0.03)	0.74 (±0.03)	0.69(±0.04)
Adjuncts		0.66 (±0.04)	0.68 (±0.04)

**Table 6:** Question–answering accuracy in Experiment 2b. Numbers in parentheses are standard errors.

	Zero interveners	One intervener	Two interveners
Arguments	0.62 (±0.05)	0.70 (±0.05)	0.7 (±0.05)
Adjuncts		0.73 (±0.05)	0.68 (±0.04)

**Table 7:** Support for/inconsistency with predictions of different theories.

	No overall SRC > ORC preference	Experimental result			
		RC-global locality pref. w/marked rel. pronouns in Expt 1	SRC bias for *chto* in Experiment 1b	Difficulty pattern at RC verb in Expt 1 and RC verb complex in Expt 2	Fronted accusative NP penalty in Expts 1 and 2
DLT	✓	✓	–	✓	?
Cue-based retrieval	✓	✓	–	✓	?
Word-order theories	(✓)	✓	✓(?)	?	?
Surprisal	(✓)	✓	✓	✗	✓
ERH	?	?	?	✗	?
Perspective shift	✗	–	✓(?)	–	–
Structural Subject Preference	✗	–	✓(?)	–	–

✓: theory makes a clear prediction of an effect matched by data; (✓): theory makes prediction that an effect (SRC > ORC asymmetry) will be weaker than seen in other studied languages, and lack of any observed asymmetry is weakly consistent with predictions; ✗: theory makes a clear prediction which is disconfirmed by the experimental result; —: theory makes no prediction; ✓(?): theory is vague in some respect but seems to make prediction consistent with empirical data; ?: theory needs further formal clarification to determine whether predictions are consistent or inconsistent with observed data.

**Fig. B.9:**
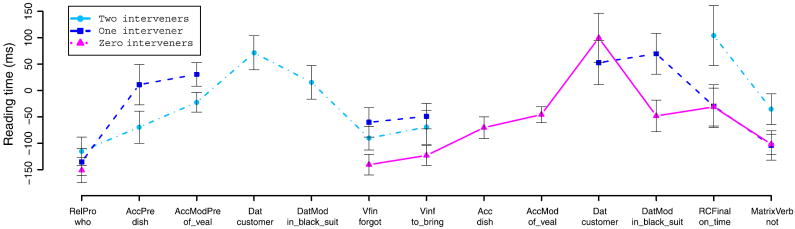
Reading times for arguments manipulation of Experiment 2a.

**Fig. B.10:**
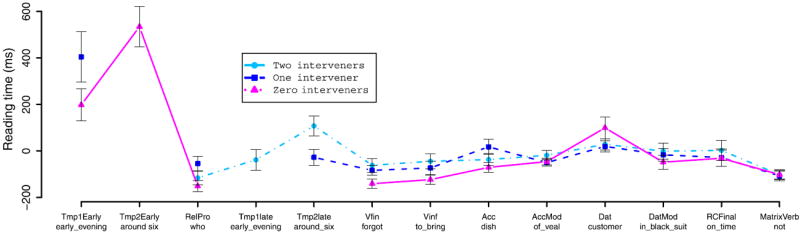
Reading times for adjuncts manipulation of Experiment 2a.

**Fig. B.11:**
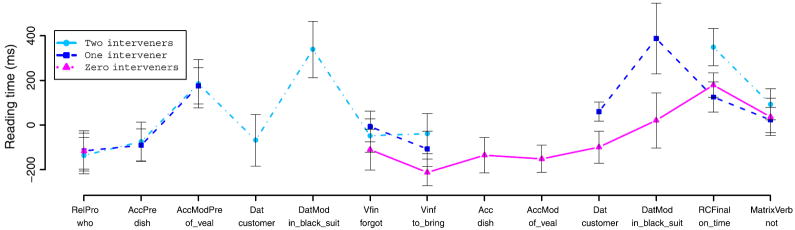
Reading times for arguments manipulation of Experiment 2b.

**Fig. B.12:**
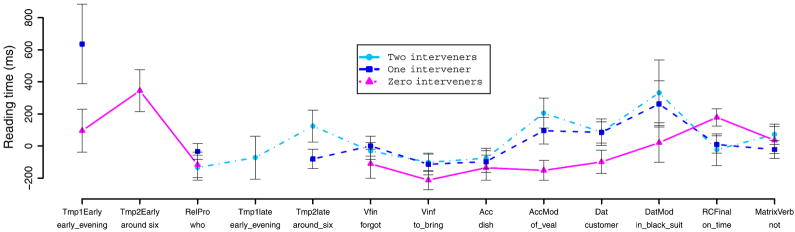
Reading times for adjuncts manipulation of Experiment 2b.

## Footnotes

1 In surprisal, this average benefit of additional preceding material can be mathematically proven: it is equivalent to the well-known proof that conditionalizing a random variable X on another random variable Y can never increase entropy (Cover & Thomas, 1991, chap. 2): *H*(*X*∣*Y*) ≤ *H*(*X*).

2 Additional preceding material that is not dependent on the verb can also create a similar contrast. For example, contrast the English ORC *the farmer who the girl with the telescope spotted* with the ORC *the farmer who the girl spotted*; the former example has an additional PP, *with the telescope*, preceding the RC verb. Even though this preceding PP is dependent on the NP subject, not on the RC verb, the semantic content of the PP nevertheless provides a useful clue as to the RC verb’s identity, and so expectation-based theories predict that the PP gives a processing benefit at the RC verb (assuming that adding the PP would in fact increase the likelihood that the RC continues with *spotted*, as measurable, for example, in a Cloze study). Nevertheless, in this paper we focus on experimental manipulations involving the preceding dependents of a verb, because they elicit a stronger contrast in the predictions of expectation-based and memory-based theories: on memory-based theories such as DLT and activation & cue-based retrieval, the predicted difficulty increase from additional preceding dependents is greater than from additional preceding non-dependent material, because the additional preverbal dependents not only interfere, they must also themselves be integrated with the verb.

3 The studies reported here are to our knowledge the first work on the online comprehension of Russian relative clauses. However, since the first presentation of Experiments 1a, 1b, and 2a, Maria Polinsky has communicated to us closely related work carried out on comprehension of SRCs and ORCs with scrambled and default word order (i.e., the same experimental design we use in Experiment 1) among monolingual child, monolingual adult, heritage child, and heritage adult speakers of Russian, testing speakers’ ability to match reversible action pictures to SRC and ORC descriptions (Polinsky, 2011). Among all but heritage adult speakers, comprehension was high across the board; among heritage adult speakers, SRC comprehension was highly accurate but ORC comprehension was at chance.

4 We refer to ORCs with SV and VS internal word orders as “default” and “scrambled” respectively for purposes of characterizing our experimental design on the logic that if the default independent-clause word order of Russian is SVO, then SV word order is obtained for an ORC by extracting the object out of a default-order transitive clause. See the next section, on Information structure and word order in Russian relative clauses, however, for a caveat as to how much should be read into the “default/scrambled” distinction theoretically.

5 *Kotoryj* is actually nominative/accusative case-syncretized for masculine and neuter inanimate head nouns, but since our experiments always involve animate head nouns we consider it “case-marking” for our purposes.

6 It should be mentioned that, with the comma absent, there is an alternative local interpretation in Russian of bigrams of the form [Noun.masculine + kotorogo] as meaning “whose Noun”; although this interpretation is not consistent with sentence-initial context, locally coherent syntactic interpretations of word sequences have been shown to affect online comprehension (Gibson, 2006; Konieczny, 2005; Tabor et al., 2004). However, there is no evidence that comprehenders entertained this possibility.

7 For example, little to no work has been done to distinguish the following views. Spillover might reflect a kind of “entrenchment” effect in which slow reading time on region n directly causes subsequent regions to tend to be read more slowly. Alternatively, spillover might reflect a “buffered processing” effect in which, when region *n* is difficult to process, only some or perhaps even none of the required processing effort happens at region *n*, so that the bulk of the RT inflation associated with region *n* is observed at region *n* + 1 and/or further downstream. The spillover analysis of our appendix addresses the first possibility, whereas the argumentation in the above paragraph addresses the second. Smith and Levy (2013) provide evidence supporting a “buffered processing” view for surprisal effects in self-paced reading, but this does not rule out the possibility that both views are simultaneously correct and/or that different types of processing difficulty behave differently with respect to spillover.

8 Though this pattern did not reach significance in Staub’s analysis, the first author of the present paper has replicated it at *p* < 0.05 in an unpublished self-paced reading study.

## Appendix B. Spillover analysis of Experiment 2

The materials of Experiment 2 were designed to minimize the possibility that any possible surprisal-based processing difficulty upon encountering preverbal accusative or dative NP arguments would spill over to the RC verb complex, by introducing additional regions postmodifying each of these NPs to “absorb” the spillover (see Experiment 2 of Grodner & Gibson (2005) for a similar design). Nevertheless, there is no guarantee that these regions are sufficient to capture all such spillover, nor is the material immediately preceding either the RC verb complex or the accusative NP—both regions showing results of critical theoretical interest—identical across condition. We therefore linear mixed models to conduct analyses of reading-time effects at these conditions attempting to account for these possible effects of spillover. There are a number of ways one might attempt to model spillover effects (e.g., Jaeger et al., 2008a; Mitchell, 1984; Vasishth & Lewis, 2006), and conducting an exhaustive comparison of possible approaches is beyond the scope of the present paper. The approach we took here can be thought of a relatively straightforward extension of standard practices for computing length-adjusted residual reading times (Trueswell et al., 1994). For each of the two experiments (2a and 2b), we fit mixed linear regression models to each non-sentence-final word of the filler materials where the length of the current word wn and the RTs of each of up to five preceding words were included as predictors, both fixed and by-subjects random effects. For each experiment we attempted to fit a set of thirty models comprised of these predictors subject to the following constraints: (i) Word length must be included as both a fixed and a random effect; (ii) If RT_*n-k*_ was to be included a fixed (respectively random) effect in the regression regression, then RT_*i*_ for all *n* − *k* + 1 < *i* < *n* had to be included as a fixed (respectively random) effect as well; (iii) An “item” random effect was included indexing either specific filler-sentence/word-number combination (fine-grained item effect) or filler sentence (coarse-grained item effect). (iv) The model must converge and be non-singular. Of the converging, non-singular models we obtained, we picked the one with the best Bayesian Information Criterion for each experiment. This gave us 4-back fixed, 1-back random effects for Experiment 2a, and 2-back fixed, 1-back random effects for Experiment 2b; in both experiments fine-grained item effects were preferred. The final resulting word-length/spillover estimated model for Experiment 2a was as follows (random-effects tables are standard-deviation/correlation matrices):

Fixed effects		
	Estimate	Std. error

(Intercept)	246.288	21.900
wordlen	31.808	3.656
rt.1back	0.150	0.018
rt.2back	0.077	0.010
rt.3back	0.044	0.009
rt.4back	0.040	0.009

Participant random effects			
	(Intercept)	wordlen	rt.1back

(Intercept)	60.535	−0.019	−0.256
wordlen	−0.019	16.367	−0.447
rt.1back	−0.256	−0.447	0.079

Word-token random effects	
	(Intercept)

(Intercept)	92.041

and the final model for Experiment 2b was as follows:

Fixed effects		
	Estimate	Std. error

(Intercept)	329.102	32.485
wordlen	47.573	16.241
rt.1back	0.043	0.013
rt.2back	0.003	0.007

Participant random effects			
	(Intercept)	wordlen	rt.1back

(Intercept)	113.093	−0.672	−0.480
wordlen	−0.672	79.437	−0.326
rt.1back	−0.480	−0.326	0.034

Word-token random effects	
	(Intercept)

(Intercept)	145.47

Note that although we see spillover effects for both experiments, there is considerably more spillover in Experiment 2a—e.g., a 15% effect from the previous word—than for 2b (a 4.3% effect).

For each of these models, we extracted the fixed-effect parameter estimates plus the conditional modes of the subject-specific random effects (the “best linear unbiased predictors”) to derive subject-specific models of word-length and spillover effects. We used these models to derive RT predictions for each word in our experimental materials (using actual, not predicted, previous-word RTs in each prediction). For each word in our experimental materials, the residual between this prediction and the true recorded RT then became the response measure for LME analyses based on our experimental manipulation, using maximal random effects structures and likelihood-ratio tests (treating twice the log of the likelihood ratio as approximately normally distributed; Baayen et al., 2008; Barr et al., 2013). The qualitative patterns at the theoretically critical Vfin, Vinf, Acc, and AccMod regions are quite similar to those shown in the main body of the paper; region-by-region reading times are shown in Figs. B.9, B.10, B.11, B.12. For the Acc and AccMod conditions we merged the one- and two-intervener conditions, as they had not diverged from one another at this point within the sentence. For Experiment 2a, these analyses in the argument manipulation yielded a significant effect at the Vfin region (*p* < 0.025) and a marginal effect at the AccMod region *p* = 0.052) but no significant effects at the Vinf or the AccMod region; in the adjunct manipulation they yielded a significant effect at the Vinf (*p* < 0.05) but not at the Vfin region. For Experiment 2b, these analyses in the argument manipulation yielded a significant effect at the finite verb (*p* < 0.01) and a significant effect at the AccMod region (*p* < 0.025), but not at the Vinf verb or Acc region; in the adjunct manipulation they yielded a significant effect at the finite verb (*p* < 0.025) but not at the non-finite verb. Finally, we conducted a meta-analysis in which we combined the residual-RT data from both experiments and included a fixed effect of experiment in the mixed models. In the argument manipulation this meta-analysis yielded significant effects at the Vfin (*p* < 0.001) and the AccMod regions (*p* < 0.01), but not at the Acc or Vinf regions. Although we do not claim that this analysis gives a definitive treatment of the problem of spillover in our experiments, it provides at least some evidence that the theoretically critical reading-time effects recovered in Experiment 2 are not obviously due to spillover effects.

## Appendix C. Materials for Experiment 1a

Only the subject-extracted, default (SVO) word order, variant “A” version of each item is given; the other versions can be constructed straightforwardly on the pattern of Example 4 in Section “Experiment 1a”, which is constructed from Item (29).

## Appendix D. Materials for Experiment 1b

These materials are highly similar to those for Experiment 1a; the key differences are that in (i) in 1a, commas were used to delimit RC onsets and offsets, but in 1b these commas were omitted; (ii) in 1a an adjunct phrase was included at the end of the RC, but in 1b there was no such adjunct phrase; and (iii) in 1a every RC NP had to serve equally as a potential head NP (see experiment design in Section “Experiment 1a”) but this constraint was not present in 1b, so different NPs and main-clause predicates were used across the two experiments in a few cases.

An example item is given below in all eight conditions; further items are given only in the subject-extracted RC, default (SVO) word order, case-marked relative pronoun condition, from which the other conditions can be derived.

## Appendix E. Materials for Experiment 2

We only present the maximally local conditions (11a and 12a) of each item. Underscores denote word sequences presented as a single region. The two temporal phrases are the last regions in the first clause, the dative argument starts at the dative noun and all following regions up to but not including the last region in the RC, and the accusative argument starts at the accusative noun and includes everything before the dative noun.
